# A Comparison of the Occurrence of Bioactive Compounds in the Grain of Different *Triticum* Species

**DOI:** 10.3390/molecules31040667

**Published:** 2026-02-14

**Authors:** Iwona Kowalska, Derya Koçak Yanik, Grzegorz Jóźwiak, Mariola Kozłowska

**Affiliations:** 1 Department of Phytochemistry, Institute of Soil Science and Plant Cultivation, State Research Institute, 24-100 Puławy, Poland; 2 Department of Food Engineering, Faculty of Agriculture, Eskişehir Osmangazi University, Eskişehir 26160, Türkiye; derya.kocakyanik@ogu.edu.tr; 3 Department of Inorganic Chemistry, Medical University of Lublin, Chodźki 4a, 20-093 Lublin, Poland; grzegorz.jozwiak@umlub.pl; 4 Department of Chemistry, Institute of Food Science, Warsaw University of Life Sciences, 02-776 Warsaw, Poland; mariola_kozlowska@sggw.edu.pl

**Keywords:** *Triticum* species, wheat grain, bioactive compounds, phenolic acids, flavonoids, alkylresorcinols, benzoxazinoids, carotenoids, tocopherols

## Abstract

This review is a collection of information on bioactive compounds found in the grain of different *Triticum* species, both old and modern. The whole wheat grain, as well as its parts, e.g., bran, contains compounds, such as phenolic acids, flavonoids, alkylresorcinols, benzoxazinoids, tocopherols, carotenoids, and others. These compounds differ in both their chemical structure and biological properties. There are significant differences in the content and composition of these compounds between *Triticum* species. Apart from the wheat species, there are many other factors influencing these differences, e.g., cultivars, environmental factors, growth conditions, and farming systems. The *Triticum* species the best researched and described in terms of the content of bioactive compounds are *Triticum aestivum* L., *T. durum* Desf., *T. spelta* L., *T. turgidum* L., *T. monococcum* L., *T. dicoccum* Schrank, *T. timopheevii*, and *T. polonicum* L.

## 1. Introduction

Bioactive compounds present in wheat grains have a significant impact on human health by reducing the risk of serious chronic diseases. Wheat grains are an important dietary source of bioactive compounds, which include, e.g., phenolic acids (PAs), flavonoids, alkylresorcinols (ARs), benzoxazinoids (BXs) [1], tocopherols, carotenoids, and other miscellaneous compounds, like phytosterols and steryl ferulates [2]. It is difficult to compare the current quantitative results with previous ones because scientists study different *Triticum* species and cultivars (cv.), use different methods of pre-treatment and determination of individual compounds, analyze different parts of wheat (whole grain, flour, bran, husks, leaves, bones, etc.), and use different solvents and methods of extraction of free phenols [3]. Published literature has documented that bioactive phytochemicals are not uniformly distributed throughout the wheat grain.

Phenolic acids (PAs) represent a large group of bioactive compounds in wheat grain, occurring in various forms [4,5,6]. Their content depends on many factors [4,7,8,9,10,11,12,13,14,15,16,17,18,19,20,21,22]. PAs play a significant role in plant defense mechanisms regardless of the type of stressors [23], in the potential positive effects of a diet rich in whole grains, especially wheat, and provide health benefits associated with a significantly reduced risk of developing chronic diseases. PAs play an important role in immune mechanisms as components of cell walls, as constitutive antibacterial compounds in plants, or induced in response to infections that cause many diseases, in particular fusarium head blight caused by *Fusarium* species. Another very important group of bioactive compounds in wheat grain are flavonoids. They are a diverse group of secondary plant metabolites that play a crucial role in plant physiology, stress adaptation, and human nutrition [24]. Their presence in wheat contributes to increased antioxidant potential, disease resistance, and potential health benefits in the human diet [25]. Furthermore, they enhance plant drought tolerance by regulating the synthesis of antioxidants and phytohormones effectively [26]. Flavonoids may also play an important role by supporting wheat’s resistance to powdery mildew infection by reducing peroxidase activity and malondialdehyde content or by reducing catalase activity to maintain ROS balance and homeostasis to support the defense-related signaling cascade [27]. Understanding their occurrence, distribution, and regulation is essential for crop improvement and increased nutritional value, especially since wheat species exhibit complex evolutionary histories and genome compositions that shape their phytochemical profiles. Alkylresorcinols (ARs), also known as resorcinolic lipids, are found in wheat grain, too. ARs’ accumulation is influenced by both genetic and environmental factors. They are found mainly in the bran [28], e.g., the outer layers of wheat grains [29]. This is why they are used as the biomarkers for whole grain wheat and play an important role in preventing and inhibiting cancer in humans [28,30]. ARs are present in wheat bran, and although the total AR content often fluctuates considerably, the distribution of AR homologs is relatively stable within species, with a C17:0/C21:0 ratio of ~0.01 for durum wheat and ~0.1 for soft wheat and spelt [28]. Another class of natural products widely distributed in wheat, concentrated in the seed coat, are benzoxazinoids (BXs) [31]. They occur in wheat in different forms [32,33,34]. BXs play a major part in the interaction and communication among plants, microbes, and insects, as well as in the formation of resistance mechanisms and quality traits of plants. Their composition and content largely depend on *Triticum* species, cultivar, environmental factors, and developmental stages [35]. Vitamin E and carotenoids are important groups of bioactive compounds present in wheat grains, too. Both are lipophilic secondary metabolites of plants. Carotenoids are popular for their health-promoting effects, such as being precursors of vitamin A, acting as an antioxidant, and reducing the risk of cancer and cardiovascular diseases [36]. They are also well-known natural pigments responsible for the yellow, orange, and red colors of plants, bacteria, fungi, and some animal tissues [37]. Moreover, this pigment influences the color of the end products and is an important quality criterion for some wheat-derived foods, such as breads, noodles, pasta, etc. On the other hand, vitamin E is known as a powerful chain-breaking natural antioxidant that cannot be synthesized in animal cells but can be found in both plants and animals. Although the clinical evidence remains inconsistent and often contradictory, vitamin E may offer a number of other health-promoting activities, such as reducing the risk of cardiovascular diseases, preventing hypertension, improving immunity, maintaining the normal metabolism of the central nervous system, increasing fertility, retarding aging, etc. [38,39,40,41]. Therefore, sustainable and adequate intake of these compounds is critical for being healthy. In this respect, wheat and wheat-based foods can be considered as the most affordable natural sources of these valuable compounds due to wheat being a widely cultivated and consumed crop throughout the world. The carotenoids and vitamin E profile in wheat species vary significantly in both composition and concentration. Even within one species of wheat, many different genotypes exist, giving rise to variations in phytochemicals. It is well recognized that the cultivar, the growing conditions, or their interaction can influence the phytochemical content of wheat [42,43]. Moreover, the various forms of vitamin E present in whole wheat may differ in their stability during storage and processing. Therefore, it is important to compare and summarize the content of bioactive compounds in different *Triticum* species.

**Figure 1:**
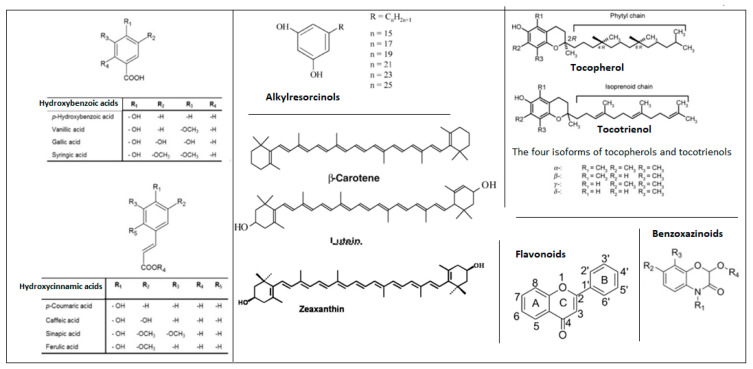
Representative structures of key bioactive compound classes in *Triticum* species.

To facilitate a clearer understanding of the structural diversity of the major bioactive compound families discussed in this review, a schematic overview of their representative chemical structures is provided (Figure 1). This scheme highlights the characteristic core structures of phenolic acids, flavonoids, alkylresorcinols, benzoxazinoids, tocols, and carotenoids, which underlie their distinct physicochemical properties and biological activities.

This review is structured following a thematic and comparative approach rather than a historical one. Each group of bioactive compounds is introduced by outlining its general chemical characteristics and biological relevance, followed by a comparative discussion of reported concentration ranges and distribution patterns across different *Triticum* species. Particular emphasis is placed on highlighting similarities and differences among species based on recent literature data. Although the health-promoting properties of wheat bioactive compounds are well established, a comprehensive and structured comparison of their distribution across different *Triticum* species remains fragmented in the literature. The objective of this review is to provide a comprehensive and comparative overview of the major bioactive compound classes present in different *Triticum* species, with particular emphasis on their qualitative and quantitative variability. By synthesizing dispersed quantitative data into a comparative perspective, this review aims to identify consistent compositional patterns, highlight variability drivers, and support future strategies in wheat breeding, crop adaptation, and the development of cereal-based foods with enhanced functional potential.

## 2. Phenolic Acids (Hydroxybenzoic and Hydroxycinnamic Acid Derivatives)

PAs are present in wheat in two forms, characterized as hydroxybenzoic and hydroxycinnamic acids [4,5,6]. Hydroxybenzoic acids are represented by vanillic (VAN), *p*-hydroxybenzoic (POH), protocatechuic (PRO), syringic (SYR), gallic (GAL), and salicylic (SAL) acids, while the hydroxycinnamic acids include ferulic (FER), caffeic (CAF), *p*-coumaric (PCO), and sinapic (SIN) acids. PAs occur in both free and bound forms. Many authors have shown that there are significant differences in PA content and composition between *Triticum* species. Differences in PA content are influenced by genetic structure, environmental factors [4], species, climate, location [7,8,9], soil composition [10,11], weather conditions [12], year of growth, habitat, plant storage conditions, drying processes, extraction methods [10], and solvents [13,14]. PA content is determined by genetic factors as well as genetic interactions with the environment, which creates differences between wheat species and cultivars within the same family [15]. The concentration of PAs depends on various agricultural practices, too [16,17]. Laddomada et al. [18] have shown that heat stress increases the accumulation of small amounts of PA, while severe drought causes an increase in FER and total phenolic acid content. It has also been found that the content of individual PAs (e.g., PCO) shows a negative correlation with some yield-related traits, including grain morphology, thousand-grain weight, and test weight [19]. Some studies have concluded that the content of PAs is related to genotype [20,21]. Wheat exposed to biotic stress contained the highest amounts of free PAs [22].

FER is the most common PA in wheat grains at all stages of wheat development [44,45,46,47,48], representing up to 90% of the total polyphenol content in wheat [49,50,51]. Okarter et al. [52] reported that the bound fraction contributed 87–97% to the total FER content. It has shown antioxidant [53], anti-inflammatory, antihypertensive, and anti-arteriosclerotic effects in vitro and in vivo [54]. The content of FER was higher in soft wheat (*T. aestivum*) than in hard wheat [21]. Serpen et al. [55] measured higher values of bound FER in emmer (*T. dicoccum* ssp. *dicoccum* Schrank.) than in einkorn (*T. monococcum* spp. *monococcum*). Several authors reported that the total FER content ranged from 64.4% to 84% [56] and 85.3% [45] of the total PA content in wheat bran, from 83.5% to 89.5% in soft wheat grain, and over 90% in wheat flour [57].

Some studies have demonstrated that grain (milling) fractions (bran, bread flour, reduction flour, and shorts) possess different content of PAs and antioxidant capacities [58]. Wheat bran is an important source of PAs (Table 1). They are considered to be a significant factor contributing to the reported protective effect of whole grain cereal consumption against chronic diseases [59]. PAs are mainly found in three layers of bran (testa, pericarp, and aleurone). They are the most numerous phenolic compounds in whole grain cereals [48]. FER, VAN, and SYR were the main PAs in the wheat bran [60]. In other studies, PAs, such as chlorogenic (CHL), CAF, PRO, PCO, and GAL, have been found in wheat bran, too [4,60,61]. López-Perea et al. [15] identified GAL, CAF, PCO, and FER in wheat bran. The type of solvent used during extraction had an impact on the differences in PA content. The chromatographic profile indicated that wheat bran had a higher concentration of CAF, ranging from 90 to 976 μg/g. The concentrations of GAL and FER were 166–181 μg/g and 91–97 μg/g, respectively.

Lower PCO, CAF, and FER contents in spring wheat bran were also shown by Pasha et al. [58]. The main PA was FER, the content of which was in the range of 137.3–180.3 μg/g. Vaher et al. [4] found a higher FER content in winter wheat bran (532 μg/g) than in spring wheat bran (269 μg/g). Both contents were higher than those found in the study by López-Perea et al. [15]. Caffeic acid 3-*O*-glucuronide, ferulic acid 4-*O*-glucuronide, FER, and PCO were identified in wheat bran of durum wheat (*T. turgidum* subsp. *durum* Desf.) by Bey et al. [3]. In wheat bran, CAF, GAL, PRO, PCO, SYR, CHL, FER, and SIN were identified by Yadav et al. [78], whereas *trans*-PCO, POH, VAN, *trans*-FER, and *cis*-FER were identified in *T. aestivum* L. by Parker et al. [79]. In einkorn and emmer wheat bran samples, PA contents were analyzed, too [80]. FER was also found to be the main PA, with content ranging from 717.9 to 1592.8 μg/g. The levels of SAL ranged from not detected (n.d.) to 389.2 μg/g. Furthermore, 2-hydroxycinnamic, *t*-FER, PCO, CAF, and VAN were detected in the ranges 211.8–586.8, 50.0–338.8, 54.4–176.0, not detected to 211.8, and 27.4–58.4 μg/g, respectively.

Whole wheat grains are also an excellent source of PAs. Typical PAs found in whole grains are FER, VAN, CAF, SYR, and PCO [81]. Among these, FER dominates, especially in its bound form, which is considered to be the main factor responsible for antioxidant activity [7,60,82]. Benincasa et al. [83] showed that einkorn and emmer wheat grains contain twice as much bound PA compared to spelt, soft wheat, and durum wheat. This was mainly due to the high content of PCO, which accounted for about 50% of the total amount of bound PA. Li et al. [8] observed a total PA content similar to that of winter, spring, and durum wheat, slightly higher than that of spelt, but slightly lower than that of emmer. However, Abdel-Aal and Rabalski [84] found higher PA content in einkorn than in several primitive and modern *Triticum* species. According to Li et al. [8], the PA content in wheat species ranged from 326 to 1171 μg/g dry matter (DM). In the study of Brandolini et al. [85], *T. monococcum*, *T. turgidum*, and *T. aestivum* grains were researched. The total content of free PA in whole grain flour was between 36.0 and 52.6 μg/g DM. The total content of bound PA varied from 441 to 715 μg/g DM, which corresponds to the results (208–964 μg/g DM) reported by Li et al. [8]. Kurasiak-Popowska et al. [86] analyzed different *Triticum* genotypes. Significant differences in content of PAs between genotypes were observed. Hidalgo and Brandolini [87] tested PA content of einkorn. The concentrations of total bound PAs varied between 484.2 and 579.5 μg/g DM. Suchowilska et al. [25] studied the content of phenolic compounds in grains of four *Triticum* species: *T. polonicum* (Polish wheat), *T. durum*, *T. aestivum*, and *T. turanicum* (Table 1). *T. turanicum*, which is closely associated with *T. polonicum*, was especially rich in POH, CHL, FER, GAL, and *t*-cinnamic acids (*t*-CIN). The FER concentration in *T. turanicum* was almost 2.7 times higher than in *T. aestivum* and almost twice as high as in *T. polonicum.* FER was the dominant PA in the grain of all four *Triticum* species, accounting for 53.4% (*T. durum*) to 62.1% (*T. turanicum*) of the total PA content. These results are lower than those reported by Horvat et al. [88] for bread wheat grain. In Suchowilska et al.’s study [25], *T. turanicum* grain had the highest content of FER, as well as four other PAs: POH, CHL, GAL, and t-CIN. *T. polonicum* differed considerably from the remaining *Triticum* species despite the low proportions of these PAs in total bound PAs (PCO 0.7% and SYR 2.9%). Karakas et al. [13] evaluated the composition of PAs (PCO, CAF, *t*-FER, and SIN) in four different wheat species, too: bread wheat (*T. aestivum* L.), durum (*T. durum* Desf.), einkorn (*T. monococcum* spp. *monococcum*), and emmer (*T. dicoccum* ssp. *dicoccum* Schrank.). Wheat species differed significantly in the PA content (Table 1). Differences in PA content in wheat grain can be attributed not only to genotypic and environmental factors, but also to differences in sample preparation procedures and determination methods. PAs occur in wheat grain primarily in bound forms and require hydrolysis for their full quantification. Factors influencing the varying PA content within the same wheat species include location, cultivar selection, and the chromatographic methods used (LC-ESI-MS/MS, UHPLC-DAD-MS, UPLC-PDA-ESI-MS, and UHPLC-MS/MS). The observed variability in PA content in the analyzed *Triticum* species is closely related to the analytical techniques used in individual studies. High-resolution mass spectrometry techniques allow for the detection of a much broader spectrum of PAs. This is confirmed by studies, e.g., on the best-described species, *T. astivum* [2,9,13,25,62,63,64,65,66,67,68,69,70] (Table 1). Five hydroxybenzoic (PRO, POH, VAN, SYR, and SAL) and four hydroxycinnamic acid derivatives (CAF, PCO, FER, and SIN) have been identified and quantified in the grain of winter wheat (*T. aestivum* L. subsp. *aestivum*) from eight European locations [9]. Other studies have shown that the total PA content in wheat genotypes grown in Hungary varied from 326 to 1171 μg/g DM [8]. Studies performed by Mpofu et al. [7] indicated that wheat genotypes grown at four locations in Canada exhibited high levels of total PA content. In Spaggiari et al.’s study [68], free and bound PA profiles were quantified in six different *Triticum* species: durum (*T. turgidum* subsp. *durum* Desf.), bread wheat (*T. aestivum* L.), turanicum wheat (*T. turgidum* subsp. *turanicum*), einkorn (*T. monococcum* L.), emmer (*T. turgidum* subsp. *dicoccum*), and spelt (*T. aestivum* subsp. *Spelta*; Table 1). The FER was the most abundant compound, representing between 67% and 73% of total PAs. The milling process led to a significant reduction in PAs. The dominant PAs were *t*-FER and SIN, as previously reported by Moore et al. [46]. Free *t*-FER was the most abundant compound in all *Triticum* species, except *T. turanicum*, in which SIN was present at a higher concentration. PCO was quantified in durum and bread wheat; in other whole grains, its content was below the limit of quantification (0.05 μg/g). Furthermore, in emmer, einkorn, and spelt, the content of free CAF was significantly higher than in durum, bread wheat, and turanicum. Total PA content was represented by the sum of free and bound forms of these compounds. The highest total PAs was obtained from bread wheat (1209.3 μg/g DM), followed by durum wheat (811.4 μg/g DM). However, no significant differences were found between turanicum (608.1 μg/g DM), einkorn (596.4 μg/g DM), spelt (568.6 μg/g DM), and emmer (508.3 μg/g DM). These findings are consistent with the studies conducted by Brandolini et al. [85], in which they performed a two-year evaluation of PA composition on einkorn, emmer, durum, and bread wheat. Since FER was the most common PA found in wheat [52], its total content in whole grains was similar and ranged between 89.5% (emmer) and 93% (bread wheat), as reported by Brandolini et al. [85]. The six PAs (VAN, SYR, CAF, PCO, FER, and SIN) in the eight *Triticum* species (*T. boeticum* L. subsp. *boeticum*, *T. monococcum* L. subsp. *monococcum, T. turgidum* L. subsp. *dicoccoides, T. turgidum* L. subsp. *turgidum, T. timopheevii* Zhuk. subsp. *timopheevii, T. aestivum* L. subsp. *spelta, T. aestivum* L. subsp. *aestivum*, and *T. aestivum* L. subsp. *compactum*) were identified by Engert and Honermeier [89]. No significant distinction between the analyzed species could be observed. FER was the predominant PA with about 66%, averaging between 185 μg GAE/g (gallic acid equivalent/g) and 247 μg GAE/g. The highest concentration of FER was found in the hexaploid species (*T. aestivum*). The diploid species (*T. monococcum* and *T. boeticum*) and the tetraploid species (*T. timopheevii* and *T. turgidum*) could keep up with the hexaploid ones. The content of FER was lower compared to those indicated earlier [8,90]. The mean content of total PAs was 334.9 μg GAE/g. Einkorn wheat (*T. monococcum* L. subsp. *monococcum*) showed a mean concentration of 306.8 μg GAE/g, and emmer (*T. turgidum* L. subsp. *dicoccoides*) 368.7 μg GAE/g. PA concentrations varied widely and were comparable to those reported for bread wheat in the literature. For example, Stracke et al. [90] reported concentrations from 282 μg/g to 1262 μg/g in wheat samples, while Zhou et al. [91] analyzed the PA composition of hard red winter wheat bran extracts with low concentrations from 202.6 μg/g to 244.1 μg/g.

Nine PAs, mainly FER, PCO, and SIN, were identified and quantified in two *Triticum* species: common wheat (*Triticum aestivum* L. subsp. *aestivum*) and spelt wheat (*Triticum aestivum* L. subsp. *spelta* (L.) Thell.) [62]. The total PA content was highest in spelt wheat (1302.3 μg/g of the gran), followed by common winter wheat (1135.1 μg/g). In another study, Kowalska et al. [63] investigated the impact of silicon treatments on PA content of common wheat and of spelt wheat. *T. aestivum* L. ssp. *spelta* species stood out. Except for SIN, this species contained significantly more other acids than common wheat, e.g., PCO contained more than 15 times more than common wheat. Spelt wheat contained a significantly higher total PA content (mean 1546.4 μg/g) than the common wheat. Silicon treatments modified PA content. The grain from four *Triticum* species: modern hull-less common wheat, modern hulled spelt wheat, old hulled emmer, and old hulled einkorn, were studied by Kowalska et al. [64], too (Table 1). Common wheat grain showed the highest PA content. The husks of *T. monococcum* L. (old husks) showed a significantly increased PA content (approximately 4000 μg/g). The PA content was significantly influenced by *Triticum* species, as in the study conducted by Zrckova et al. [92]. Both in the grain and in the husk, the highest PA content was found in the species of *T. aestivum* L. ssp. *spelta* (803.8 ug/g DM) and *T. monococcum* (827.3 ug/g DM). FER was the dominant PA in the grain, while PCO dominated in the husk. Barański et al. [12] observed that the total free PA content was higher in spelt (*T. aestivum* L. ssp. *spelta*) and amounted to 599.8 μg/g DM, while in emmer (*T. dicoccum* Schrank) it amounted to 590.0 μg/g DM. Similarly, the content of bound PAs in spelt wheat was 564.6 μg/g DM, while in emmer wheat it was 555.4 μg/g DM. Spelt wheat had the highest free POH content at 2.0 μg/g DM and the highest SYR content at 3.3 μg/g DM. The average level of total PAs in einkorn was 615 μg/g DM, whereas that in the *T. monococcum* was slightly higher (779 μg/g DM). The lowest average level (579 μg/g DM) of the total PAs was observed in the spelt. A report published by Skrajda-Brdak et al. [69] showed the PA content in spring durum wheat, whereas the study of Lacko-Bartosowa et al. [65] determined and compared individual PA concentrations of emmer and common wheat. The predominant PA was FER, which accounted for 67.10% of free PAs in common wheat and up to 66.02% in emmer (Table 1). A study by Serpen et al. [55] showed that emmer had a higher total PA content than einkorn and bread wheat. Li et al. [8] found that emmer, durum, and bread wheat had the highest PA contents, but these values did not differ significantly from those for spelt and einkorn wheat [77,93]. PA contents of einkorn, bread, and durum wheat were determined by Sahin et al. [66]. FER and PCO of einkorn were significantly higher than bread and durum wheat. Similar results were observed in diploid *T. urartu* Tum. by Yilmaz et al. [77] (Table 1) and by Brandolini et al. [85] for soft red winter wheats. FER contents ranged from 455.9 to 621.5 μg/g [85], and from 25.1 to 54.2 μg/g for PCO. These values were lower than those found in einkorn populations. Moreover, the FER values were similar to those reported for emmer and einkorn species by Serpen et al. [55].

Laddomada et al. [76] analyzed seven subspecies of *T. turgidum*: subsp. *turanicum* (Jakubz.), subsp. *durum* (Desf.) Husn., subsp. *polonicum* (L.) Thell., subsp. *turgidum*, subsp. *dicoccum* (Schrank ex Schubler) Thell., subsp. *carthlicum* (Nevski), and subsp. *dicoccoides* (Korn. ex Asch. et Graebner) Thell. They showed that durum wheat had the highest average concentration of total PAs. The lowest concentration of PAs was found in *T. turgidum* subsp. *dicoccum* (Schrank ex Schübler) Thell. (Table 1). In another study, Laddomada et al. [18] showed the effect of elevated temperatures and water deficit on the PA profile of the durum wheat (*Triticum turgidum* ssp. *durum* (Desf.) Husnot). Severe heat stress increased the accumulation of PAs such as PCO, SYR, and VAN, and decreased the accumulation of major PAs (e.g., FER), while severe drought had a greater effect on FER and total PAs. In Italian durum wheat (*T. turgidum* ssp. *durum* (Desf.)), PAs were determined by Menga et al. [19]. PAs were extracted from whole grain flour. FER was the most abundant PA (438.3 µg/g DM), followed by PCO, SIN, VAN, SYR, and POH. Bellato et al. [94] reported PA levels ranging from 98.6 to 144.9 µg/g DM in *T. durum* wheat. In another study, Menga et al. [11], in *T. durum* wheat, obtained an average PA content of 882 µg/g DM. Rachoń et al. [73] investigated the effect of two different levels of cultivation technology, medium and high, on the PA content in the grain of four species of winter wheat: durum (*T. turgidum* ssp. *durum*), common wheat (*T. aestivum* ssp. *vulgare*), spelt (*T. aestivum* ssp. *spelta*), and einkorn (*T. monococcum*). Einkorn had the highest content of PAs. Common wheat and spelt responded with a decrease in the content of the acids when tested using the higher level of cultivation technology, while their content increased in durum wheat and einkorn (Table 1). Falcinelli et al. [95] measured PA content in *T. aestivum* L. grains cultivated in four different nitrogen fertilization schedules. Targeted analysis using LC-MS/MS allowed for the identification of hydrobenzoic acids (CHL, PRO, POH, VAN, SYR, and ELG) and hydrocinnamic acids (FER, PCO, CAF, and SIN). The grains obtained from crops subjected to late nitrogen deficiency produced wheat with much higher PAs (as compared to the other nitrogen treatments). Hadjout et al. [53] showed in durum wheat that the content of bound PAs was significantly higher than that of free PAs. Seven free PAs (CHL, PCO, *t-*FER, SIN, SYR, PRO, and VAN) and eight bound PAs (PCO, FER, SYR, VAN, 8-5′-dehydrodiferulic, 5-5′-dehydrodiferulic, 8-O-4′-dehydrodiferulic, and 8-5′-benzofuran-dehydrodiferulic acid) were determined. In the case of acids, the main compound was FER, whose level was significantly higher (up to 4913.9 μg/g DM), followed by PCO (up to 3099.0 μg/g DM; Table 1).

The content of free, conjugated, and bound PAs in four different colored (standard red, yellow endosperm, blue aleurone, and purple pericarp) grains of wheat (*T. aestivum* L.) was analyzed by Paznocht et al. [72]. Five PAs (FER, SIN, PCO, VAN, and POH) and *cis*-isomers of FER and SIN were determined. The total PA content of colored wheat groups varied: blue aleurone > purple pericarp > yellow endosperm > red color (798 > 702 > 693 > 599 μg/g). FER prevailed in red and yellow wheats, VAN in blue, and PCO in purple wheats [96]. Liu et al. [97] examined the PA content in red, yellow, purple, and common wheat. Purple wheat contained the highest FER content (814 μg/g). PAs were also studied in bread, durum, and colored wheat by Dinelli et al. [98], Liu et al. [97], Nicoletti et al. [99], and Sharma et al. [100]. A study by Zhang et al. [101] showed that the outer bran of blue wheat had the highest total bound PAs (3458.7 µg/g), while the short bran of purple wheat had the lowest value (1730.7 µg/g). Ma et al. [102] reported that among PAs, the levels of bound FER, VAN, and CAF were significantly higher in purple wheat than in white and red wheat, whereas the levels of total soluble PAs, soluble FER, and VAN were significantly higher in purple and red wheat than in white wheat. GAL, PRO, POH, ELG, SYR, PCO, *m*-CO, *o*-CO, FER, CAF, SIN, and CHL were detected in the grain of 40 purple wheat (*T. aestivum*) genotypes by Shamanin et al. [67]. Benzoic acid derivatives and GAL were dominant in purple wheat samples (Table 1).

## 3. Flavonoids in *Triticum* Species

Flavonoids, as well as related groups of phenolic compounds, such as coumarins, stilbenes, and lignans, are important bioactive components found in cereal grains, including wheat grain, mainly in the seed coat and embryo, both in free and bound forms (esters, glycosides, and cell-wall-bound compounds). Flavonoids found in wheat grains are mostly flavones containing the aglycone groups of apigenin, luteolin, and chrysoeriol, as well as flavonols with kaempferol or quercetin as the skeleton structure. The flavonoid content depends on the species, cultivar, and grain color (pericarp/aleurone), as well as environmental factors and the analytical methods used to identify them. These methods often differ in specificity and sensitivity, which affects the comparability of the results obtained.

In the grains of winter wheat (*T. aestivum* L.), the presence of luteolin-8-*C*-glucoside (orientin) and luteolin-6-*C*-glucoside (isoorientin) was detected. These compounds exhibited higher expression levels in mature grains than in immature ones. Moreover, a total of 81 flavonoids and 10 lignans were identified in developing wheat grains. The higher accumulation of phenolic compounds could be due to upregulated structural and regulatory genes [103]. Some ancient *Triticum* species, such as einkorn (*T. monococcum*) and emmer (*T. turgidum* ssp. *dicoccum*), may produce lower yields than modern common and durum wheat, and they often exceed them in terms of flavonoid content and overall nutritional value. They also exhibit higher levels of phenolic compounds, including FER, and antioxidant activity compared to common and durum wheat [104]. Samples of emmer and einkorn wheat may also be characterized by a higher content of bound than free forms of flavonoids, amounting to 37.3 (einkorn) and 24.4 mg of quercetin/100 g of grain (emmer), respectively. However, Sahin et al. [66] found slight differences in flavonoid content among wheat species, such as einkorn (*T. monococcum* ssp. *monococcum*), bread wheat (*T. aestivum* L.), and durum (*T. durum* Desf.), but observed significant differences within species. Žilić et al. [105] also found no significant differences in flavonoid content between bread wheat (*T. aestivum* L.) and durum wheat (*T. durum* Desf.) species but observed differences between their individual genotypes. The flavonoid content in bread wheat ranged from 0.028 to 0.042 mg CE/g DM, and in durum wheat from 0.027 to 0.033 mg CE/g DM. A high variability was also observed between genotypes of common Italian wheat (*T. aestivum* L.). The six old wheat genotypes showed a higher number of total phenolic compounds and their isomeric forms than the modern cultivars, which may indicate that breeders should use old wheat genotypes in breeding programs aimed at developing bread wheat cultivars with a significant contribution of compounds with health-promoting properties [5].

By examining the total flavonoid content in eighteen different indigenous Turkish wheat cultivars (*T. monococcum*, *T. dicoccum*, *T. durum*, and *T. aestivum*), Akram et al. [106] showed that their content decreased from the *T. aestivum* genotype, which had the highest average value (380.4 mg CE/kg DM), through *T. durum* with an average value of 344.6 mg CE/kg DM, to the *T. monococcum* genotype with an average value of 301.0 mg CE/kg DM, and the *T. dicoccum* genotype with the lowest average value of 290.1 mg CE/kg DM. In turn, the *T. aestivum* genotype achieved a higher total flavonoid content (451.6 mg CE/kg DM). Additionally, in bread wheat, 29 of the 50 principal components were identified as flavonoids. These were among the components with the most significant variability in terms of spatial distribution within the grains. In general, wheat with medium gluten content and pigmented ones were characterized by higher levels of bioactive compounds in the outer layers of the grain than wheat with high and low gluten content, making them more valuable for the production of whole grain food products [107].

Eight flavonoids (apigenin, catechin, kaempferol, luteolin, naringenin, quercetin, rutoside, and vitexin) were identified in the grains of selected spring accessions of durum wheat and *T. durum*. Quercetin and naringenin were the dominant flavonoids, at 45.7 and 33.7 mg/kg, respectively, and the flavonoid content ranged from 113.0 to 202.0 mg/kg [50]. In studies conducted by Bianco et al. [108], using local and genetically improved cultivars of Sicilian durum wheat (*T. durum* Desf.), it was shown that local cultivars had a richer and more diverse phenolic profile than modern wheats. Among the 13 metabolites identified were flavone-*C*-glycosides, e.g., luteolin di-*C*-hexoside, apigenin *C*-hexoside-*C*-pentoside, and apigenin *C*-hexoside-*C*-hexoside *O*-glucuronide. In grain samples from Tunisian durum wheat (*T. turgidum* ssp. *durum*), the presence of apigenin-6-*C*-*β*-galactosyl-8-*C*-*β*-glucosyl-*O*-glucopyranoside and apigenin-6-*C*-arabinoside-8-*C*-hexoside (schaftoside and isoschaftoside), also belonging to flavone-*C*-glycosides, was detected using UHPLC-QTOF-MS/MS. Isoflavones were also detected and were only present in the free phenolic fractions. In addition, tricin, eupatorin, quercetin-3,7-di-*O*-glucoside, and isorhamnetin derivatives were identified in the tested wheat grains and in the bound phenolic fractions of wheat samples, as well as anhydrosecoisolariciresinol, classified as a plant lignan produced in the phenylpropanoid metabolic pathway [109].

In the study by de Camargo et al. [110], whole durum wheat was found to have a lower proportion of phenolic acids (19.3%) than apigenin derivatives quantified by LC-ESI-QTOF-MS. The concentration of these derivatives (apigenin-6-*C*-arabinoside-8-*C*-hexoside and apigenin-6-*C*-β-galactosyl-8-*C*-β-glucosyl-*O*-glucuronopyranoside), which belong to flavonoids, ranged from 63.5% to 80.7%. The apigenin di-*C*-glycosides (ACGs) occur in bread wheat and other related cereal grains, mainly as one or two sets of isomers containing arabinose and glucose (ACG1) or arabinose and galactose (ACG2) in the A ring of apigenin, and are a potential target for wheat breeders trying to develop new cultivars contributing to the yellowish color of their products, thereby limiting the use of color additives [111]. Higher concentrations of flavonoids are found in wheat cultivars with colorful grains, particularly those with purple, black, or blue hues. They are a rich source of anthocyanins, identified primarily as derivatives of cyanidin, pelargonidin, delphinidin, and peonidin. Delphinidin derivatives, such as delphinidin-3-glucoside, delphinidin-3-galactoside, and delphinidin-3-rutinoside, are responsible for the blue color of the grain, while cyanidin and pelargonidin derivatives, including cyanidin-3-glucoside, cyanidin-3-(6-malonyl glucoside), cyanidin-3-rutinoside, peonidin-3-glucoside, and peonidin-3-(6-malonylglucoside), are responsible for the purple color [112]. These compounds are mainly found in the pericarp layer and in the aleurone layer of wheat grains. Differences in their accumulation between species are largely genetically determined (specific genes determine the color of pericarp vs. aleurone) and depend on environmental factors, such as UV radiation, temperature, infection with pathogenic fungi, phytohormones, and ions. Among the species, cultivars with a blue aleurone layer (more common in bread wheat, *T. aestivum*) and purple pericarp in some forms of *T. durum* and its hybrids exhibit the highest concentrations of anthocyanins. In blue wheat (*T. aestivum* L., cv. Skorpion) and purple wheat (*T. aethiopicum* Jakubz cv. *Abyssinskaja arrasajta*), anthocyanins were identified, with a total content of 9.3 mg/kg in blue wheat and 13.2 mg/kg in purple wheat [113]. Among the bread wheat (*T. aestivum*), including purple, red, yellow, and white wheat, purple wheat had significantly the highest flavonoid content (from 21.6 to 103.0 mg of catechin equivalents/100 g DM basis) and total anthocyanin content (from 2.5 to 23.5 mg of cyanidin-3-glucoside equivalents/100 g of wheat grain). In turn, the total anthocyanin content in the remaining wheat, whose extracts were light yellow, was below 1 mg/100 g. Twenty different types of flavonoids were identified in the purple wheat cultivar, using LC-MS/MS chromatography. Most of them were flavones bearing aglycone groups of apigenin, luteolin, or chrysoeriol, and a few belonged to flavonols with kaempferol or quercetin as the backbone in the form of glycoconjugates [97]. Differences in the anthocyanin content of wheat grain can be attributed not only to genotypic and environmental factors but also to differences in extraction procedures and analytical protocols. Anthocyanins in wheat occur predominantly as polar glycosylated compounds, often acylated, and are localized mainly in the pericarp or aleurone layers, which makes their determination particularly sensitive to sample preparation conditions. One of the key factors affecting the efficiency of anthocyanin extraction is the solvent’s polarity. Most commonly, aqueous alcohol mixtures (e.g., methanol–water or ethanol–water systems) are applied, usually acidified to enhance solubility and stabilize the flavylium cation of anthocyanins [97]. However, variations in solvent composition may result in selective extraction of specific anthocyanin derivatives and co-extraction of other phenolic compounds, thereby affecting both quantitative determination and chromatographic separation. In addition, the use of higher acid concentrations or elevated extraction temperatures may lead to partial degradation or structural transformations, particularly of acylated anthocyanins, which may undergo deacylation or decomposition in response to pH changes. In contrast to phenolic acids, which occur in wheat grain mainly in bound forms and require hydrolysis for complete quantitative determination, anthocyanins are chemically labile and susceptible to hydrolytic conditions. Acid hydrolysis may result in cleavage of glycosidic moieties followed by degradation of anthocyanidins, leading to alterations in anthocyanin profiles or underestimation of their total content. Therefore, direct comparison of results obtained using different extraction approaches (with or without hydrolysis) may be misleading. An additional source of discrepancy is the analytical technique used. Spectrophotometric methods provide total anthocyanin content values expressed as equivalents, whereas chromatographic techniques (HPLC/UPLC/LC-MS) enable the identification of specific compounds but are highly dependent on extraction efficiency, the availability of standards, and matrix effects specific to cereal samples [113]. Consequently, standardization of extraction procedures and clear specification of whether intact glycosylated anthocyanins or their transformation products are being quantified are essential for result comparability and proper data interpretation.

Pigmented wheat cultivars, such as black wheat and green wheat, showed significantly higher anthocyanin levels than common wheat, which contained only low levels of peonidin-3-(6-*O*-*p*-coumaroyl)-glucoside. Black wheat contained high levels of cyanidin-3-*O*-(6-*O*-malonyl-β-D-glucoside) (23.2 μg/g), cyanidin-3-*O*-glucoside (6.5 μg/g), and peonidin-3-*O*-glucoside (2.3 μg/g), of which cyanidin-3-*O*-glucoside is particularly known for its anti-inflammatory and antioxidant properties [114]. In studies conducted on *Triticum* species (*T. polonicum, T. durum, T. aestivum*, and *T. turanicum*) whose grains were light yellow or golden yellow in color, no statistically significant differences in flavonoid content were observed [25]. The species studied were characterized by the presence of eight flavonoids, such as apigenin, luteolin, quercetin, kaempferol, catechin, naringenin, rutin, and vitexin (Table 2). Significant differences were found only in the mean rutin concentrations in all milling fractions and grains. Its concentration was the lowest in *T. turanicum* grains, flour, and bran, amounting to 6.3, 4.0, and 14.8 mg/kg, respectively. In contrast, *T. turanicum* wheat grains and milling fractions were characterized by high concentrations of quercetin, naringenin, and vitexin. The quercetin concentration in *T. turanicum* grains (104.8 mg/kg) was more than five times higher than in bread wheat (19.6 mg/kg) and more than twice as high as in Polish wheat (44.1 mg/kg).

Nine flavonoids, including dihydroquercetin, vitexin, dihydrokaempferol, rutin, quercitrin, quercetin, naringenin, kaempferol, and apigenin, were identified in the sprouts of eight wheat cultivars planted and harvested in China. Three of them, namely, dihydroquercetin, quercitrin, and vitexin, were the main representatives of this group of phenolic compounds, with the total content of dihydroquercetin showing an increasing tendency with increasing germination time. Compared with wheat grain, an increase in the content of three flavonoids, dihydrokaempferol, naringin, and kaempferol, was observed in germinating wheat. The increase in the content of these compounds was associated with changes in gene expression and enzyme activity (PAL, C4H, and 4CL), which are key to the biosynthesis pathway of phenolic substances [115]. The combination of UPLC-PDA-ESI/HRMSn and MDF techniques allowed for the identification of 72 flavone *C*-glycosyl derivatives in wheat sprouts (*T. aestivum* L.), including 2 mono-*C*-glycosides, 34 di-*C*-glycosides, 14 acyl di-*C*-glycosides, and 7 acyl tri-*C*-glycosides, which were considered important fractions containing marker compounds in the differentiation of whole grain and refined products. Additional compounds identified were acylated mono-*O*-glycosyl-di-*C*-glycosyl flavones and acylated di-*C*-glycosyl flavones [116]. In turn, eleven flavonoid *C*-glycosides have been found in Oriental wheat (*T. turgidum* ssp. *turanicum*) sprouts, also known as Khorasan wheat or under the trade name Kamut^®^, originating from Türkiye and Afghanistan. Six of them were reported for the first time in Oriental wheat *T. turgidum* ssp. *turanicum* and two for the first time in the *Triticum* genus (chrysoeriol-8-C-glucoside-2″-O-arabinoside and chrysoeriol-8-C-glucoside-2″-O-rhamnoside) [117]. However, analysis of the chemical composition of gamma-irradiated mutant lines, an original cultivar, and a certified cultivar of wheat (*T. aestivum*) identified 14 flavonoid components, including 11 flavone *C*-glycosides, 2 flavone *O*-glycosides, and 1 flavone. In 37 tested wheat sprout samples, significant differences in content were observed with regard to 3 flavone *C*-glycosides, namely, isoschaftoside (apigenin 6-*C*-arabinoside 8-*C*-glucoside), isoorientin (luteolin 6-*C-*glucoside), and isoscoparin (crysoeriol 6-*C*-glucoside). In addition, the presence of tricin 7-*O*-malonylhexoside and tricetin trimethyl ether, and other apigenin and luteolin derivatives, was found [118]. Tricin (5,7,4′-trihydroxy-3′,5′-dimethoxyflavone) occurs in most species of monocotyledonous plants, especially in the Poaceae family, mainly in cereal crops, such as oats, barley, rice, wheat, and corn, playing a key role as a natural protective agent, acting as an antioxidant and a defense compound against biotic (fungi and pathogens) and abiotic (cold, UV radiation, drought, and salt) stresses [119]. The highest content of this compound (33.1, 32.7, and 28.0 mg/g of lignin) was found in oats, wheat, and brachypodium. It also exhibits numerous biological activities, e.g., antiallergic, antiviral, anti-inflammatory, antioxidant, immunomodulatory, and antibacterial [120]. The observed variability in flavonoid diversity and content among the analyzed wheat samples may be closely associated with the analytical techniques employed in individual studies. A targeted HPLC-DAD approach enables precise quantification of selected flavonoids using reference standards. However, this approach is often limited to a narrow set of known compounds, potentially underestimating the total flavonoid pool. In contrast, high-resolution mass spectrometry techniques (LC-MS/MS and UPLC-QTOF-MS) applied in untargeted metabolomic studies allow for the detection of a much broader spectrum of flavonoids, including numerous *C-* and *O*-glycosylated and acylated derivatives that are not efficiently detected by UV-based methods. Nevertheless, metabolomic datasets are typically based on relative signal intensities rather than absolute concentrations, which complicates direct quantitative comparisons with targeted analyses. Consequently, the observed dependence of flavonoid content on the analytical method reflects not only biological variability but also fundamental differences in detection sensitivity, selectivity, and quantification strategies. It should also be emphasized that wheat is a complex matrix rich in phenolic compounds with similar chemical properties, which may lead to interference effects. Therefore, methods with lower selectivity may lead to either underestimation or overestimation of the content of specific flavonoids.

Wheat grains also contain coumarins, stilbenes, and lignans, but usually in relatively low concentrations compared to phenolic acids and classic flavonoids. Coumarins are compounds widely distributed in the plant kingdom, produced in large quantities in the Umbelliferae, Rutaceae, Compositae, and Leguminosae plant families, performing important biological functions, for example, participating in plant defense mechanisms against pathogens and environmental stress [121]. Coumarin derivatives include esculetin, umbelliferone, and scopoletin, compounds with antioxidant, antimicrobial, and allelopathic properties. As representatives of phenolic compounds, they also occur in the grains of various *Triticum* species, primarily in free or bound forms. Still, their content is usually low and difficult to compare precisely between cultivars due to the limited data available in scientific publications. Coumarin itself can influence many physiological processes at different stages of plant growth and development, including germination, root growth, or the activity of various enzymes, e.g., by stimulating the synthesis and secretion of amylase from the aleurone layer of uniform wheat (*T. aestivum* L.) [122]. However, it would be essential to reveal the potential regulatory effects of coumarin on other plant developmental processes and its possible interactions with different plant growth regulators. The lack of comprehensive comparative studies prevents the identification of specific cultivars with the highest numerical values. However, studies conducted by Lachman et al. [123] using the grains of five cultivars of wheat (*T. aestivum* L.) demonstrated the presence of coumarin, 4-hydroxycoumarin, and, in each cultivar tested, 7-hydroxycoumarin (umbelliferone) in the range of 8.7–133.0 mg/kg DM (Table 3).

In the study by Hamli et al. [124] using durum wheat (*T. turgidum* L. var. *durum*), the presence of coumarins was also detected based on qualitative screening tests. The presence of these compounds was also confirmed in the seeds of four new Algerian wheat cultivars, including two durum wheat (*T. durum* Desf.) and two soft wheat (*T. aestivum* L.) cultivars [125]. HPLC-ESI-TOF-MS analysis allowed for the separation and preliminary identification of a total of 104 compounds in the free and bound phenolic fractions of whole grains of old and modern cultivars of common wheat from Italy (*T. aestivum* L.), among which, in addition to phenolic acids and flavonoids, stilbenes, lignans, and coumarins were also present [5].

The presence of pinosylvin derivatives, including monoglycoside and diglycoside pinosylvin, was demonstrated within the stilbenes group. These compounds were identified primarily in the bound fraction, suggesting their association with the cell wall structures of the grain’s outer layers and indicating that their content will depend on the extraction, hydrolysis, and detection methods used. In turn, a metabolomic comparison of 7 closely related *T. aestivum* lines with grains of different colors revealed the presence of 3 stilbenes (pinosylvin, polydatin, and resveratrol) among 85 identified polyphenolic compounds [126]. The occurrence of these compounds may be more pronounced in specific lines and not necessarily ‘universal’ for the entire species. Phenolic profiling of 7 different wheat (*T. aestivum*) genotypes at different stages of grain development (milky, softy, physiological maturity, and mature) revealed the presence of 237 phenolic compounds, divided into 5 classes: flavonoids (85), phenolic acids (77), other polyphenols (51), lignans (16), and stilbenes (8). It was observed that, as the grain ripened, the number of identified phenolic compounds decreased. The highest total content (free + bound) was found in immature grains, whereas the lowest was observed in mature grains, and the overall phenolic profile differed between genotypes [127].

Lignans occur in relatively small amounts in wheat grain, primarily in the seed coat and bran, and their profiles and concentrations vary significantly among wheat cultivars and types, including durum, common, spelt, and old local cultivars. The most common lignans in cereals—secoisolariciresinol, matairesinol, pinoresinol, lariciresinol, and syringaresinol—are converted by intestinal bacteria into the mammalian lignans enterodiol and enterolactone, which are subsequently absorbed in the large intestine [128]. The percentage contribution of individual lignans may vary depending on the species and genotype studied. This was demonstrated in a study of 156 cereal samples collected from 8 locations in Finland. The analyzed material included 73 samples of spring wheat representing 9 cultivars. There was a wide variation in the total content of lignans in the grain among the cultivars. For spring wheat, values ranged from 340 to 2270 µg/100 g of grain, largely due to genetic differences [129]. Kim et al. [130] also reported that the total lignan content in most of the cereal grains analyzed was relatively low, including that of wheat. In their study, no lignans were detected in wheat grain except for syringaresinol. Similarly, Dinelli et al. [131] demonstrated the presence of secoisolariciresinol and pinoresinol in common wheat (*T. aestivum*), as well as arctigenin, hinokinin, and syringaresinol, which were detected exclusively in older genotypes. These findings may indicate the potential applicability of these compounds in a wide range of both conventional and specialized food products. The tissue localization of lignans is also crucial for understanding their occurrence in grain. A substantial proportion of grain lignans (approximately 70–85%) is associated with the aleurone layer, a single cell layer located at the boundary between the endosperm and the pericarp, which is technologically classified as part of the bran fraction [132]. Consequently, interspecific comparisons among wheat species (*T. aestivum, T. durum, T. spelta, T. monococcum,* and *T. dicoccum*) should take into account both genetic differences and the fact that lignan content in grain strongly depends on whether whole grain, bran, or flour with varying degrees of milling is analyzed.

## 4. Alkylresorcinol (Resorcinolic Lipids) Content in Wheat Species

ARs are long-chain phenolic compounds similar to tocopherols, except for the presence of a straight aliphatic hydrocarbon side chain and a single phenolic ring. Five 5-*n*-alkylresorcinols (C17:0–C25:0), 5-*n*-alkylresorcinols, 5-alkenylresorcinols, 5-oxoalkylresorcinols, 5-oxoalkenylresorcinols, and 5-hydroxyalkenylresorcinols, with a saturated aliphatic chain at the C-5 position of the benzene ring, are the major AR classes in wheat. Of these, C19:0 and C21:0 are the most numerous ARs in wheat grain. Fifteen different homologues of ARs in wheat bran were identified by Cantele et al. [133], including five saturated chain: 5-*n*-heptadecylresorcinol (C17:0), 5-*n*-nonadecanylresorcinol (C19:0), 5-*n*-heneicosylresorcinol (C21:0), 5-*n*-tricosylresorcinol (C23:0), and 5-*n*-pentacosylresorcinol (C25:0), and ten monounsaturated ARs (two positional isomers for each abovementioned homolog). Martin-Garcia et al. [59] found the same AR composition in aleurone fractions of wheat bran. The composition of the homologues (saturated plus monounsaturated) was the following: C17:0, 7.5%; C19:0, 41.9%; C21:0, 40.8%; C23:0, 7.2%; C25:0, 2.6%, as already observed by another researcher [134]. The ratio between C17:0 and C21:0 was 0.18, in agreement with those reported in soft wheat [28]. Differences in the results obtained for wheat grains may result from differences between *Triticum* species, cultivars, environmental conditions, and the determination method used. Other studies involving different *Triticum* species have shown that AR accumulation is influenced by both genetic and environmental factors [29,134,135]. Zarnowski et al. [136] examined the alkyl- and alkenylresorcinol homologue composition and content in grains of soft spring and soft winter wheat (*T. aestivum* L.) as well as of hard wheat (*T. durum* Desf.), grown in Poland. Nine different resorcinolic homologues: C15:0, 5-*n*-heptadecenylresorcinol (C17:1), C17:0, 5-*n*-nonadecenylresorcinol (C19:1), C19:0, 5-*n*-heneicosenylresorcinol (C21:1), C21:0, C23:1, C23:0, and C25:0, were found in all wheat samples. The predominant ARs in wheat grains were C21:0 and C19:0. A lower total AR content was found for *T. durum* Desf., compared to *T. aestivum* L. The results show that ARs may be useful as chemotaxonomic markers for a distinction between soft and hard wheat plants. Kowalska and Jędrejek [31] analyzed AR content in a grain of spring and winter wheat (*T. aestivum*) cultivated under different production systems. The AR profile consisted of five 5-*n*-alkylresorcinol (C17:0, C19:0, C19:1, C21:0, C23:0, and C25:0) derivatives, among which C21:0 and C19:0 predominated. The highest total amount of ARs was found in organic crops compared to the conventional ones. The average concentrations of total ARs were definitely higher in winter wheat cultivars (mean 796 μg/g) than in spring ones (mean 680 μg/g). In other studies, Kowalska et al. [9] determined the concentration of ARs in winter wheat (*T. aestivum* L.), grown at eight European locations. Nine AR derivatives were identified and quantified: 5-*n*-alkylresorcinols, including C15:0, C17:0, C19:0, C21:0, C23:0, and C25:0, and 5-*n*-alkenylresorcinols, including C19:1, C21:1, and 5-*n*-heneicosadienylresorcinol (C21:2), among which C21:0 and C19:0 predominated, too. Both location and atmospheric conditions influenced the AR contents. The average total resorcinol lipid content was highest in wheat grown in Spain (954.8 μg/g), while the lowest in the United Kingdom (749.1 μg/g) and Ireland (719.9 μg/g). These results are similar to the data presented by Ross et al. [29] (Table 4). Sagi-Lomniczi and Sagi [137] showed AR content in durum wheat (*T. durum*) at the level from 460 to 1080 µg/g. Comparisons of ARs in different *Triticum* species were also made by Andersson et al. [134] (Table 4). The highest AR contents were shown in *T. spelta* grain, followed by *T. monococcum* and *T. dicoccum*. Similar AR contents were shown by Landberg et al. [138] for *T. aestivum* and *T. durum*, and by Ross et al. [139] for *T. spelta*. Five species of wheat (*T. dicoccum*, *T. monococcum*, *T. aestivum, T. durum*, and *T. turgidum*) were analyzed by Tsirivakou et al. [140]. A GC-MS analysis showed a mixture of 5-*n*-alkylresorcinols with a side chain ranging from C15 to C25, but predominantly C19 and C21. Grains of old cultivars of *T. dicoccum* and *T. monococcum* showed the highest contents of ARs. Significantly lower AR contents were found in *T. aestivum* and *T. durum* (Table 4). Studies of Ross et al. [141] also showed that *T. monococcum* and *T. dicoccum* grains contained more ARs than *T. aestivum* and *T. durum* grains. Pedrazzani et al. [142] described differences in AR content in four wheat species. Seven 5-*n*-alkylresorcinol (C17:0, C19:0, C19:1, C21:0, C21:1, C23:0, and C25:0) profiles in einkorn (*T. monococcum* spp. *monococcum*), emmer (*T. turgidum* spp. *dicoccum*), spelt (*T. aestivum* spp. *spelta*), and common wheat (*T. aestivum* spp. *aestivum*) species were determined. Common wheat had the highest total AR content, followed by emmer, spelt, and einkorn (Table 4), in agreement with a previous study of Righetti et al. [143]. *T. monococcum* spp. *monococcum* and *T. turgidum* spp. *dicoccum* are often supposed to have higher contents of ARs [138]. These data slightly exceed previous reports, which showed that the AR content in spelt, emmer, and einkorn was 580–820 µg/g DM [144]. C21:0 was definitely present in the highest content (from 44.9–60.0%). C19:0 was the second most abundant homologue in the hexaploid species *T. aestivum* spp. *aestivum* (25.3%) and *T. aestivum* spp. *spelta* (26.1%), followed by C23:0, C19:1, C17:0, C21:1, and C25:0. In contrast, emmer and einkorn contained higher portions of C23:0, that was 28.4% and 28.3%, respectively, followed by the homologues C19:0, C25:0, C21:1, and C19:1. This trend was in agreement with previously reported data [29,138,145]. In two wheat species: common wheat (*T. aestivum* L. subsp. *aestivum*) and spelt wheat (*T. aestivum* L. subsp. *spelta* (L.) Thell.), ARs were analyzed by Kowalska et al. [62]. Nine alkylresorcinols (C15:0, C17:0, C19:0, C19:1, C21:0, C21:1, C21:2, C23:0, and C25:0) were identified and determined in winter Polish grain. The main homologues were C21:0 and C19:0, too. The total AR content showed significant variability (*p* < 0.05). *Triticum aestivum* L. subsp. *spelta* contained less total ARs than *Triticum aestivum* L. subsp. *aestivum* (Table 4). In other study, Kowalska et al. [64] identified six AR derivatives (C17:0, C19:0, C19:1, C21:0, C23:0, and C25:0) in four wheat species: common wheat—*T. aestivum* L. subsp. *aestivum* (modern hull-less), spelt wheat—*T. aestivum* L. subsp. *spelta* (L.) Thell. (modern hulled), emmer—*T. dicoccum* Schrank (Schuebl), and einkorn—*T. monococcum* L. (old hulled). The highest total AR content was found in common wheat grain, followed by spelt, emmer, and einkorn. Similarly, in the husk, the highest total AR concentration was marked in spelt, followed by emmer and einkorn (Table 4). The C21:0 and C19:0 homologues were concentrated especially in wheat grains, while C25:0 and C21:0 were characteristic homologues in husks. C21:0 and C19:0 were the primary homologues of ARs in 17 different cultivars of *T. aestivum* Chines wheat grains, too. The average total AR concentration was 603 µg/g. Other AR derivatives identified included C23:0, C17:0, and C25:0 [114]. Ciccoritti et al. [146] analyzed the effect of thermal treatments on AR in *T. turgidum* L., ssp. *durum* and *T. monococcum* ssp. *monococcum* grown in Italy. AR content ranged from 136 μg/g in einkorn to 246 μg/g in durum wheat. These values were lower than those reported by Andersson et al. [134] and Ciccoritti et al. [145] who, in addition, found a higher AR content in einkorn with respect to durum wheat (600 and 362 μg/g DM, respectively).

## 5. Benzoxazinoids (BXs) in *Triticum* Species

BXs occur in wheat in three forms: benzoxazolinones, including 2-benzoxazolinone (BOA) and 6-methoxy-2-benzoxazolinone (MBOA), hydroxamic acids, including 2,4-dihydroxy-1, 4-benzoxazin-3-one (DIBOA), 2,4-dihydroxy-7-methoxy-1, and 4-benzoxazin-3-one (DIMBOA), and their corresponding 2-β-D glucosides and lactams, including 2-hydroxy-1, 4-benzoxazin-3-one (HBOA), 2-hydroxy-7-methoxy-1, 4-benzoxazin-3-one (HMBOA), and their respective 2-β-D glucosides [32,33,34]. According to Villagras et al. [147], the main BX in wheat was DIMBOA (and its glycosides). Studies conducted by Nicol et al. [148] showed that the DIMBOA concentration in 47 *Triticum* cultivars (mainly *T. aestivum*) ranged from 1 to 8 mmol/kg fresh weight (FW). According to Tanwir et al. [33], the DIMBOA concentration in wheat grains ranged from 4.8 to 95 μg/g DM. Quantitative analysis by Pihlava et al. [149] showed that, of all BX derivatives, the diglycosides HBOA-glc-hex and DIBOA-glc-hex are the most abundant components in whole grain wheat. Comparing the individual or total BX content in different literature sources is not straightforward. One reason is that many publications focus solely on the analysis of a selected group of benzoxazinones (usually DIMBOA and DIBOA), while in other studies the total benzoxazinoid content may include glycosides, other benzoxazinones, and benzoxazolinones.

Few studies indicate their presence in dry wheat grains [32,33]. Hanhineva et al. [32] conducted a detailed qualitative characterization of BXs present in whole grain wheat samples. Several novel benzoxazinoid metabolites of the hydroxamic acids (DIBOA and DIMBOA), lactams (HBOA), and benzoxazolinones (BOA) were identified, including double-hexose derivatives of DIBOA, DIMBOA, and HBOA.

Content of BXs increases during germination [2,150] but, on the other hand, is easily degraded during fermentation [151]. Zivkovic et al. [152] investigated the effect of germination on the BX composition in spelt (*T. spelta* L.) grains. Germination significantly increased the content of BXs in free and bound forms. In non-germinating wheat grains, BXs were not present, but BOA, MBOA, and HBOA were identified after 24–96 h of germination. MBOA was present in the highest amounts after 96 h of seed germination (277.6 µg/g DM). Content of BXs after two days of soaking, in Polish spring and winter cultivars of common wheat (*T. aestivum* L.), was reported by Kowalska and Jędrejek [31]. Six different BXs, including hydroxamic acids and their glycosides (DIBOA, DIMBOA, DIBOA-Glc, and DIMBOA-Glc), benzoxazolinone (MBOA) and lactam (HBOA), were determined after two days of soaking but have not been found in dry wheat seeds. The average concentrations of total BXs were definitely higher in spring wheat cultivars (mean 325 μg/g) than in winter cultivars (mean 226 μg/g). The main BXs identified in wheat cultivars were DIBOA and DIMBOA-Glc (in spring cultivars), and MBOA and DIMBOA-Glc (in winter cultivars). Similar contents of total BXs in whole grain and refined wheat samples (71–409 μg/g) were found by Dihm et al. [153]. Batyrshina et al. [154] measured the levels of BXs in young wheat plants of *T. aestivum*, *T. turgidum* ssp. *dicoccoides*, and *T. turgidum* ssp. *durum*, 11, 15, and 18 days after germination. Three BXs were detected and identified, including 2-hydroxy-4,7-dimethoxy-1,4-benzoxazin-3-one glucoside (HDMBOA-Glc), DIMBOA, and 4-dihydroxy-7,8-dimethoxy-1,4-ben-zoxazin-3-one glucoside (DIM_2_BOA-Glc). For *T. turgidum* ssp. *dicoccoides*, lower BX levels were recorded than for *T. aestivum* and *T. turgidum* ssp. *durum*. Baranzelli et al. [2] also showed that germination significantly increases the BX content in soft and hard *T. aestivum* L. Two BXs belonging to the hydroxamic acids subclass were identified, namely, DIMBOA-hex-hex and DIBOA-hex-hex. DIBOA-hex-hex was the major benzoxazinoid. The average content of DIBOA-hex-hex and DIMBOA-hex-hex in ungerminated seeds was 27 and 7 µg/g, respectively, while after 72 h of germination these values increased to 125 and 74 µg/g, respectively. Previous studies have already shown that the dihexoside DIBOA is the main BX found in wheat [32,33]. The second highest BX content was HBOA-dihexoside. This BX has been reported in wheat grains at concentrations of 0.06 µg/g [33] and, according to Pedersen et al. [155], this concentration can be increased by germination to 16 µg/g (49 lmol/kg) in wheat grains. Moreover, a methyl derivative of DIMBOA, 4,7-dimethoxy-1,4-benzoxazin-3-one-2-O-β-D-glucopyranoside (HDMBOAglc), was isolated and characterized from *T. aestivum* germ [156]. Besides, Nakagawa et al. [157] detected hydroxamic acid glucosides and aglucones in wheat seeds after wetting and before germination, but not in dry seeds.

BXs have been reported to be particularly abundant in the germ of wheat grains, which is part of the bran fraction after grain milling. Tanwir et al. [33] analyzed seed fractions obtained from wheat milling. Significantly higher concentrations of these bioactive compounds were found in the germ than in the remaining fractions, e.g., bran and endosperm. The dominant compounds in the various wheat fractions were the dihexoses 2,4-dihydroxy-1,4-benzoxazin-3-one (DIBOA-glc-hexose) and 2-hydroxy-1,4-benzoxazin-3-one (HBOA-glc-hexose). Twelve BXs were identified in the grain. The dominant BX in wheat grain was DIBOA-glc-hex (3.29 µg/g DM), followed by HBOA-glc-hex (0.47 µg/g DM), DIMBOA (0.22 µg/g DM), DIBOA (0.14 µg/g DM), HMBOA-glc, and DIBOA-glc (0.12 µg/g DM). Nine BX metabolites in the embryo samples and seven BX metabolites in the endosperm samples were identified in *T. turgidum* ssp. *dicoccoides*, *T. turgidum* ssp. *dicoccum*, and *T. turgidum* ssp. *durum* by Ben-Abu and Itsko [158]. Higher concentrations of BX derivatives, such as HBOA, BOA, DIBOA, and DIMBOA, were found in both the embryo and the endosperm of *T. turgidum* ssp. *dicoccum* and *T. turgidum* ssp. *durum*. Pedersen et al. [155] reported that whole wheat flour from *T. durum* did not contain BXs, and pure wheat flour contained only trace amounts of HMBOA-Glc. Other studies, similar to the present study, failed to detect Bxs in dry wheat seeds [159,160]. Hydroxamic acid (Hx) contents of 17 different *Triticum* species were analyzed by Niemeyer [161]. Hx was found in all wheat species tested. The highest Hx values were found in *T. speltoides* (16.0 mmol/kg FW), then in *T. turgidum* (6.4 mmol/kg FW), *T. polonicum* (6.2 mmol/kg FW), *T. dicoccum* (6.1 mmol/kg FW), *T. durum* (4.9 mmol/kg FW), and the lowest in *T. tauschii* (0.2 mmol/kg FW).

## 6. Comparative Profile of Tocols in *Triticum* Species

The vitamin E profiles in *Triticum* species differ significantly in both composition and concentration. Vitamin E in cereals consists of tocopherols (T) and tocotrienols (T3), collectively known as tocols. Naturally occurring vitamin E molecules can exist in eight different forms depending on the number and position of methyl groups in the chromanol ring (α-3 CH_3_, β-2 CH_3_, γ-2 CH_3_, and δ-1 CH_3_) and varying saturation of the prenyl group (Figure 1). Tocopherols contain fully saturated tails, while tocotrienol tails contain three trans double bonds in the prenyl group [162]. The tocotrienols represent the predominant form of vitamin E found in cereal grains, while animal-derived foods serve as abundant sources of α-tocopherol. Wheat grains typically contain both forms, with tocotrienols accounting for approximately 60–80% of total tocols. The α-types of tocopherols and tocotrienols are regarded as the forms with the highest metabolic activity. Recent studies also suggest that tocotrienols exhibit stronger antioxidant properties compared to tocopherols [163]. However, despite their greater ability to scavenge free radicals, tocotrienols are absorbed less efficiently in the body [164]. The tocotrienol/tocopherol ratio (T3/T) is a useful indicator of the nutritional and functional profile of wheat vitamin E. A higher ratio usually corresponds to enhanced antioxidant potential and is, therefore, of interest in assessing nutritional quality. There are separate reports and data in the literature regarding tocopherol and tocotrienol content in some common wheat species, showing large differences. For instance, in recent years, Suriano et al. [165] compared the tocols in different genotypes of *T. turgidum* ssp. *durum*, *T. turgidum* ssp. *dicoccum*, *T. aestivum* ssp. *aestivum*, and *T. aestivum* ssp. *spelta*. According to the results of this study, the average total tocol content of *T. dicoccum* was the lowest (25.05 μg/g DM), while *T. aestivum* ssp. *spelta* showed the highest content (34.78 μg/g DM). The average total tocol content of *T. aestivum* ssp. *aestivum* was moderate (28.46 μg/g DM). This result is in agreement with total tocol content in spring wheat (*T. aestivum* L.) investigated by Hejtmánková et al. [166]. However, it was too low compared to an average tocol content of the *T. aestivum* group (62.75 μg/g DM) reported by Hidalgo et al. [167]. The tocotrienols (*β* + *γ*) was reported as the most abundant individual tocol. The *α*-tocopherol concentration of *T. turgidum* and *T. aestivum* species varied between 2.91 and 10.65 μg/g DM. It was also reported that *T. aestivum* ssp. *spelta* exhibited the highest value with 10.65 μg/g DM. The range of *α*-tocopherol concentration was almost similar, as reported by Moore et al. [46] for different *T. aestivum* L. genotypes. The average *α*-tocopherol concentration of *T. turgidum* ssp. *durum* genotypes was 6.05 μg/g DM, and this value was also comparable to the average *α*-tocopherol content (7.91 μg/g DM) of durum wheat studied by Žilić et al. [168]. However, the *α*-tocopherol contents were lower than those reported for different durum wheat cultivars (8.34–12.55 μg/g DM) by Hidalgo et al. [167] and by Lampi et al. (10.7 μg/g DM) [169]. In addition, Suriano et al. [165] emphasized that there were significant differences between genotypes in all groups in terms of both individual tocols and the tocotrienols/tocopherols ratio.

In another study, Lachman et al. [170] studied tocols of einkorn (*T. monococcum* L. subsp. *monococcum*), emmer (*T. dicoccum* subsp. *dicoccum*), and spring wheat (*T. aestivum* L.). Einkorn contained the highest total amount of tocol (average 56 μg/g DM,), followed by emmer (average 41.33 μg/g DM) and spring wheat (average 36 μg/g DM), respectively. These values are in agreement with the total tocol content of emmer (40.92 μg/g DM for fall sowing) and einkorn (53.6 μg/g DM for fall sowing) reported by Giambanelli et al. [43]. However, the total tocol content reported for einkorn was lower than those reported by Hidalgo et al. [167]. On the other hand, the average total tocol content of emmer in this study was higher than those reported for wild emmer *T. dicoccum* Schübl. (Schrank) (19.7–22.7 μg/g DM) by Hejtmánková et al. [166]. The *β*-tocotrienol was the most abundant tocol among the other tocols at almost 30 μg/g DM on average in einkorn wheats. Similarly, *β*-tocotrienol was also reported as the most abundant in einkorn by Hidalgo et al. [167]. However, Hidalgo et al. [167] reported a higher total tocol value (48.2 μg/g DM) than that obtained by Lachman et al. [170]. The *α*-tocopherol content of einkorn was reported in the range of 8.1–17.4 μg/g DM, while the *α*-tocopherol contents of non-einkorn wheat controls, *T. dicoccum* and *T. aestivum*, were reported at, on average, 9.6 and 14.4 μg/g DM, respectively.

Hussain et al. [42] investigated tocopherol and tocotrienol contents of organically grown wheat genotypes. They belonged to landraces, old cultivars, modern cultivars, spelt wheat, and primitive wheat. Total tocol contents were in the ranges of 27.5–36.5, 23.7–37.3, 32.0–32.9, 23.1–34.8, and 21.9–35.2 μg/g DM for the genotypic groups of landraces, old cultivars, modern cultivars, spelt, and primitive wheat, respectively. These results were in similar ranges as recently reported by Suriano et al. [165] for *T. turgidum* ssp. *durum*, *T. turgidum* ssp. *dicoccum*, *T. aestivum* ssp. *aestivum*, and *T. aestivum* ssp. *spelta.* However, they were much lower than previously reported by Lachman et al. [170] for einkorn, emmer, and spring wheat, by Lampi et al. [169] for winter, spring, durum, and spelt, and by Hidalgo et al. [167] for einkorn, *T. turgidum*, and *T. aestivum*. As it was in a previous study, *β*-tocotrienol content was the highest among the tocols. The *α*-tocopherol content was in the range of 5.2–10.3 μg/g DM. Giambanelli et al. [43] compared the bioactive content of einkorn (*T. monococcum* L., subsp. *monococcum*), emmer (*T. turgidum* L., subsp. *dicoccum*), Zanduri wheat (*T. timopheevii* subsp. *timopheevii*), Georgian emmer (*T. turgidum L*., subsp. *paleocolchicum*), durum wheat (*T. turgidum* L., subsp. *durum*), macha wheat (*T. aestivum* L., subsp. *macha*), and bread wheat (*T. aestivum* L., subsp. *aestivum*). The total tocol content ranged between 26.6 and 72.8 μg/g DM with an average of 55.0 μg/g DM. The primitive genotype exhibited a similar average value as the studied ones. The predominant tocol was *β*-tocotrienol among all studied species. *T. monococcum* and the tetraploid (*T. timopheevi* var. *rubiginosum*) and the hexaploid (*T. macha*) exhibited higher (>3.5) tocotrienol/tocopherol ratios. The tocol contents of the primitive genotypes were comparable to those of modern cultivars, contrary to the prevailing presumption that primitive forms contain higher levels of bioactive compounds. Whent et al. [171] compared the tocopherol compositions of whole wheat flours from white and red wheat cultivars. The *α*-tocopherol contents of the wheat flours were reported as 46.9, 12.2, 17.3, 61.0, and 20.9 μg/g, for Blanca Grande, Alpowa, Louise, WestBred 936, and Macon, respectively. These levels are strikingly high and much higher than values typically reported in European and Asian cultivars. The *α*-tocopherol content was much higher than those reported for some other wheat cultivars by Lampi et al. [169], Lachman et al. [170], Hidalgo et al. [167], Suriano et al. [165], and Moore et al. [46]. Hidalgo et al. [167] studied the change in tocols in einkorn (*T. monococcum* ssp. *monococcum* L.), *T. turgidum* (ssp. *durum* and *dicoccum*), and *T. aestivum* (ssp. *aestivum* and *spelta*) as a control for comparison. The total tocol content of the controls was reported as an average of 52.9 and 62.8 μg/g DM for *T. turgidum* and *T. aestivum*, respectively, and an average of 78.0 μg/g DM for einkorn. The most abundant tocol was reported as *β*-tocotrienol (48.2 μg/g DM), followed by *α*-tocotrienol (12.8 μg/g DM), *α*-tocopherol (12.2 μg/g DM), and *β*-tocopherol (4.8 μg/g DM) in einkorn. It can be concluded that the total tocol content of einkorn significantly exceeding the non-einkorn wheats (*T. turgidum* and *T. aestivum*).

Lampi et al. [169] examined the vitamin E profile—focusing on tocopherols and tocotrienols—across a wide range of wheat species. They analyzed bread wheat (*T. aestivum* var. *aestivum*), durum wheat (*T. turgidum* var. *durum*), spelt (*T. aestivum* var. *spelta*), as well as einkorn (*T. monococcum* var. *monococcum*) and tetraploid emmer (*T. turgidum* var. *dicoccum*). The average total tocol contents of winter, spring, and durum wheat and spelt were 49.9, 49.6, 48.1, and 46.2 μg/g DM, respectively. Einkorn once again exhibited the highest mean content (57 µg/g), while emmer displayed the lowest (36.4 µg/g). The widest variation was observed in the winter wheat species, which contained both the genotype with the lowest concentration (27.6 μg/g DM) and the one with the highest concentration (79.7 μg/g DM). As noted in previous studies [165,167,170], *β*-tocotrienol was the dominant tocol, with the exception of a single winter wheat cultivar. The *α*-tocopherol contents were reported as 7.7, 9.1, 10.7, 11, 13.5, and 13.9 μg/g DM for tetraploid emmer, diploid einkorn, durum wheat, spelt, winter wheat, and spring wheat, respectively. Lv et al. [172] investigated soft red winter wheat (*T. aestivum* L.). *α*-Tocopherol was detected in all wheat flour samples, with concentrations ranging from 0.1 to 0.9 µg/g of flour, representing a 7.7-fold variation (*p* < 0.05). These concentrations were lower than those reported by Moore et al. [46], who examined eight Maryland-grown soft red winter wheat cultivars. The observed differences may be attributable to the removal of the bran layer during sample preparation. Moore et al. [46] studied selected Maryland-grown soft red winter wheat (*T. aestivum* L.). They reported almost 3-fold differences at concentrations of *α*-tocopherol among the studied cultivars. The *α*-tocopherol concentration ranged between 3.4 and10.1 µg/g. Žilić et al. [105] also investigated the tocopherol content in wholemeal bread (*T. aestivum* L.) and durum (*T. durum* Desf.) novel wheat species. They examined the *α*- and *β* + *γ*-tocopherol contents of each bread and durum wheat and reported that *α*-tocopherol content changed in the ranges of 4.3–9.2 µg/g DM and 4.7–10.2 µg/g DM, for bread and durum wheat, respectively. Similarly, they found that, overall, there were significant differences among the bread and durum wheat. However, the mean *α*-tocopherol levels in bread wheat (6.5 µg/g DM) and durum wheat (7.9 µg/g DM) did not differ significantly.

## 7. Comparative Profile of Carotenoids in *Triticum* Species

The carotenoids profile in wheat species varies significantly in both composition and concentration. Wheat species, particularly durum wheat and specialty wheats like einkorn, Khorasan, and Kamut, are important sources of carotenoids, mainly lutein and zeaxanthin [36]. Lutein and zeaxanthin are hydroxy-containing carotenoids that do not serve as provitamin A but are essential for supporting eye health and help in anti-aging and reduce the risk of cardiovascular diseases and cancer [173,174,175]. Hence, carotenoid content of wheat is important in the prediction of the nutritional value of products made from wheat grains. Therefore, the carotenoid content of different wheat species has been compared in this study. While individual studies report a wide range of concentrations, a consolidated overview of the most consistent trends across *Triticum* species is provided in Table 5, which summarizes the key quantitative patterns highlighted throughout this review. Table 5 summarizes the carotenoid compositions of wheat species reported in the literature in recent years. As seen here, carotenoid present in wheat can differ by cultivar. Lutein was the dominant carotenoid in all species (80–87% of total carotenoids), while the levels of zeaxanthin, *α*-carotene, *β*-cryptoxanthin, and *β*-carotene were found in lower amounts. Wheat species showed substantial variability in carotenoid composition, with einkorn (*T. monococcum*) consistently exhibiting the highest concentrations of lutein and total carotenoids, typically ranging from 5.3 to 13.6 µg/g [69,87]. Durum wheat (*T. turgidum* ssp. *durum*) also displayed elevated levels, with lutein commonly between 3 and 6 µg/g and total carotenoids reaching 7–8 µg/g in some studies [25,36,165]. Emmer (*T. dicoccum*) and spelt (*T. spelta*) showed intermediate values, with emmer typically containing 2–6 µg/g and spelt 3–4 µg/g total carotenoids [69,87,165]. In contrast, common bread wheat (*T. aestivum*) generally exhibited the lowest carotenoid content, often below 2 µg/g in modern cultivars, though genotypes selected for yellow endosperm may reach 6.8 µg/g [87,165,176]. Other ancient or regional species, such as *T. polonicum, T. turanicum*, and *T. timopheevi*, possess moderate carotenoid levels (3–5 µg/g), higher than most bread wheats but lower than einkorn and durum [25,43].

## 8. Influence of Genotype, Environment, and Agronomic Practices on Bioactive Compounds in Wheat

In addition to species-specific differences, the concentration and composition of bioactive compounds in wheat grains are strongly influenced by several external factors, including cultivar, environmental conditions, growth stage, and agronomic practices. Numerous studies have demonstrated that genetic background plays a decisive role in determining phenolic profiles, particularly for phenolic acids and flavonoids [85,86,87]. Environmental factors, such as temperature, water availability, and soil composition, further modulate phytochemical accumulation, often through stress-induced metabolic responses [19,73]. Moreover, farming systems, including conventional versus organic cultivation, fertilization regimes, and technological input levels, have been reported to significantly affect the levels of phenolic acids, carotenoids, and tocols in wheat grain [53,94,95]. These interacting factors contribute substantially to the wide variability reported in the literature and should, therefore, be considered when comparing bioactive compound data across different *Triticum* species. Moreover, the distribution of these compounds among wheat fractions is highly heterogeneous, with bran and germ generally showing higher concentrations compared to the endosperm. Such variability highlights the importance of considering both genetic and processing-related factors when interpreting compositional data across wheat species.

## 9. Conclusions

Overall, the available data demonstrate substantial diversity in phenolic acids, flavonoids, alkylresorcinols, benzoxazinoids, tocopherols, and carotenoids content among *Triticum* species. Literature data indicate that *T. aestivum* L., *T. durum* Desf., *T. spelta* L., and *T. monococcum* L. are rich sources of PAs; however, wheat husks and bran contain the highest amounts of these acids. The dominant acid in the grain of all *Triticum* species is FER. Higher concentrations of flavonoids, mainly anthocyanins, are found in wheat with colorful grains. Grains of old cultivars of *T. dicoccum* and *T. monococcum* showed the highest contents of ARs. The C21:0 and C19:0 homologues are concentrated especially in wheat grains, while C25:0 and C21:0 are characteristic homologues in wheat husks. BXs are particularly abundant in the germ of wheat grains, and their content increases during germination. The main BX in wheat is DIMBOA (and its glycosides). *T. monococcum* L. exhibits the highest total tocols, followed by emmer, spelt, and modern bread wheat. Durum wheat and spelt also contain appreciable levels of tocotrienols, which typically constitute the majority of vitamin E in wheat. The predominance of *β*-tocotrienol across all species highlights its important contribution to antioxidant potential. The results indicate that einkorn includes 2–4 times more lutein than non-einkorn wheats and durum wheat is the richest carotenoid source, whereas modern bread wheat generally contains the lowest levels. Considerable intra-species variation suggests that both genetic background and environmental conditions influence phytochemical content. Beyond summarizing compositional differences, the comparative analysis presented in this review highlights that the variability of bioactive compounds among *Triticum* species is the result of complex interactions between genetic background, environmental conditions, and agronomic practices.

Such knowledge can be harnessed to guide breeding programs aimed at enhancing bioactive compound profiles, to optimize cultivation practices under diverse environmental conditions, and to support the development of wheat-based foods with targeted functional properties.

## Figures and Tables

**Table 1:** PA content (μg/g) in different *Triticum* species.

*Triticum* Species	Material	Hydroxycinnamic Acids				Hydroxybenzoic Acids						Total	References
		FER	CAF	PCO	SIN	VAN	POH	PRO	SYR	GAL	SAL		
*T. aestivum* L. subsp. *aestivum*	grain	544.2	86.7	7.7	66.9	20.8	49.1	30.3	11.1	9.3	n.r.	n.r.	[25]
		3400.0	≤0.02	0.77	≤0.005	n.r.	n.r.	n.r.	n.r.	n.r.	n.r.	4170	[13]
		20.4	-	10.6	7.8	13.7	13.1	n.r.	18.9	n.r.	n.r.	84.0	[2]
		684.9–817.8	17.4–22.0	14.9–23.0	46.3–73.1	18.7–23.4	1.9–3.5	3.1–4.5	13.0–20.7	n.r.	1.1–1.4	821–961	[9]
		710.4–929.6	26.8–45.0	34.2–50.3	38.1–65.5	20.4–27.8	2.1–5.4	4.6–6.8	11.1–26.7	n.r.	1.5–1.8	879–1135	[62]
		729.9–795.4	26.8–30.9	22.2–28.8	89.0–99.8	26.0–31.5	5.6–8.9	3.2–3.3	21.5–28.9	n.r.	1.7–1.8	937–1018	[63]
		524.9–1063.0	9.1–21.5	7.2–38.6	31.6–83.1	5.7–10.3	2.8–9.8	1.1–2.0	4.8–11.7	n.r.	LOD	599–1223	[64]
		19.9	0.6	1.1	2.1	n.r.	1.5	n.r.	3.3	n.r.	1.1	396	[65]
		148.7–554.8	n.r.	5.1–33.5	n.r.	n.r.	n.r.	n.r.	n.r.	n.r.	n.r.	n.r.	[66]
		4.0–14.3 (f.f.) 1.5–582.0 (b.f.)	4.2–6.7 (f.f.) n.d.–5.6 (b.f.)	0.9–5.3 (f.f.) 0.3–23.9 (b.f.)	2.8–28.8 (f.f.) 0.2–618.9 (b.f.)	n.r.	n.d.–7.7 (f.f.) n.d.–1.4 (b.f.)	6.9–66.4 (f.f.) 2.6–160.8 (b.f.)	n.d.–5.8 (f.f.) n.d.–3.0 (b.f.)	5.4–95.3 (f.f.) n.d.–29.1 (b.f.)	n.r.	n.r.	[67]
		1.9 (f.f.) 564.2 (b.f.)	0.3 (f.f.) 1.2 (b.f.)	0.1 (f.f.) 5.0 (b.f.)	1.2 (f.f.) 40.3 (b.f.)	n.r.	0.1 (f.f.) 2.3 (b.f.)	n.r.	n.r.	n.r.	n.r.	1209.3	[68]
		489.4	n.r.	15.2	20.7	5.0	6.0	n.r.	1.9	n.r.	n.r.	538.2	[69]
		25.4	17.7	n.r.	n.r.	n.r.	n.r.	n.r.	n.r.	n.r.	n.r.	283.4	[70]
	bran	51.9	67.3	n.r.	n.r.	n.r.	n.r.	n.r.	n.r.	n.r.	n.r.	n.r.	[70]
		1195.2	190.2	17.5	146.4	46.4	109.1	66.2	24.4	20.5	n.r.	n.r.	[25]
		31.9–74.2 (f.f.) 2550–3650 (b.f.)	6.9–24.3 (f.f.) 46.0–186.0 (b.f.)	6.0–16.9 (f.f.) 58.4–101.0 (b.f.)	n.r.	44.9–211.0 (f.f.) 34.2–104.0 (b.f.)	1.2–5.0 (f.f.) 42.6–153.0 (b.f.)	168.0–656.0 (b.f)	12.9–35.6 (f.f.) 21.3–98.1 (b.f.)	n.r.	n.r.	n.r.	[71]
		137.3–180.3	0.9–1.5	24.0–32.0	n.r.	n.r.	n.r.	n.r.	55.3-94.0	n.r.	n.r.	n.r.	[72]
		9.5 (f.f.) 1330.8 (b.f.)	0.8 (f.f.) 4.9 (b.f.)	0.1 (f.f.) 16.9 (b.f.)	7.9 (f.f.) 123.8 (b.f.)	n.r.	0.5 (f.f.) 11.7 (b.f.)	n.r.	n.r.	n.r.	n.r.	n.r.	[68]
*T. aestivum L.* subsp. *spelta* (L.) Thell.	grain	884.0	43.9	264.9	34.1	42.7	7.0	6.7	17.2	n.r.	1.9	1302.4	[62]
		921.3	29.8	422.9	60.2	50.6	12.6	5.2	41.0	n.r.	2.9	1546.4	[63]
		614.9	10.2	11.4	51.8	6.9	5.7	1.5	8.6	n.r.	LOD	711.1	[64]
		5292–7442	235–441	497–635	823–1080	n.r.	603–683	n.r.	79–106	n.r.	n.r.	9790–13,730	[73]
		1.3 (f.f.) 300.6 (b.f.)	0.4 (f.f.) 0.8 (b.f.)	0.1 (f.f.) 5.2 (b.f.)	0.8 (f.f.) 22.8 (b.f.)	n.r.	0.1 (f.f.) 1.6 (b.f.)	n.r.	n.r.	n.r.	n.r.	568.6	[68]
		1.1–41.5 (f.f.) 132.5–667.9 (b.f.)	0.2–2.7 (f.f.) 2.1–6.2 (b.f.)	0.0–0.8 (f.f.) 0.8–13.6 (b.f.)	0.6–5.9 (f.f.) 76.5–445.4 (b.f.)	0.1–1.4 (f.f.) 1.5–3.6 (b.f.)	0.3–13.4 (f.f.) 5.0–16.2 (b.f.)	0.8–86.5 (f.f.) 0.4–16.8 (b.f.)	1.0–50.1 (f.f.) 0.5–3.0 (b.f.)	0.0–16.3 (f.f.) 0.0–78.5 (b.f.)	0.0–12.7 (f.f.) 0.0.-3.0 (b.f.)	507–1257	[74]
		412.6	n.r.	9.6	17.8	4.4	5.2	n.r.	1.9	n.r.	n.r.	451.5	[69]
		4.7 (f.f.) 1696.2 (b.f.)	1.0 (f.f.) 7.5 (b.f.)	0.1 (f.f.) 21.0 (b.f.)	3.2 (f.f.) 115.4 (b.f.)	n.r.	0.7 (f.f.) 9.1 (b.f.)	n.r.	n.r.	n.r.	n.r.	n.r.	[68]
		1871.3	35.4	1691.2	7.7	32.2	15.0	4.8	31.7	n.r.	2.6	3062–4319	[64]
	bran	5056–7042	116–432	231–557	744–1087	n.r.	219–753	n.r.	32–98	n.r.	n.r.	9160–11,890	[73]
	husk	749.9	132.3	4.0	70.5	23.6	104.2	46.1	27.4	9.5	n.r.	n.r.	[25]
*T. aestivum* ssp. *vulgare*	grain	2080.0	≤0.02	0.60	≤0.005	n.r.	n.r.	n.r.	n.r.	n.r.	n.r.	2680	[13]
*T. durum* Desf.	grain	397.0–480.1	n.r.	11.3–23.8	n.r.	n.r.	n.r.	n.r.	n.r.	n.r.	n.r.	n.r.	[66]
		367.4	n.r.	9.0	32.1	5.1	4.8	n.r.	1.9	n.r.	n.r.	420.3	[69]
		43.3–56.7 (f.f.) 2916.4–4913.9 (b.f.)	n.r.	5.1–6.6 (f.f.) 1601.7–3099.0 (b.f.)	6.4–28.0 (f.f.)	3.5–4.6 (f.f.) 16.2–32.2 (b.f.)	n.r.	1.1–1.5 (f.f.)	3.2–4.5 (f.f.) 16.5–27.1 (b.f.)	n.r.	n.r.	n.r.	[53]
		1949.9	352.6	10.8	187.9	61.9	276.4	121.1	75.6	25.3	n.r.	n.r.	[25]
		2.3 (f.f.) 1169.6 (b.f.)	n.r.	0.7 (f.f.) 43.6 (b.f.)	0.6 (f.f.) 26.4 (b.f.)	1.3 (f.f.) 15.0 (b.f.)	7.9 (b.f.)	n.r.	0.5 (f.f.) 8.4 (b.f.)	n.r.	n.r.	n.r.	[75]
	bran	663.8	n.r.	25.0	131.3	6.7	2.3	n.r.	2.7	n.r.	n.r.	832	[76]
*T. turgidum* subsp. *durum* (Desf.)	grain	563.1	n.r.	14.5	53.2	8.8	4.3	n.r.	5.7	n.r.	n.r.	649.9	[18]
		4652–7315	114–218	355–800	565–1193	n.r.	417–457	n.r.	40–48	n.r.	n.r.	10,270–13,730	[73]
		1.7 (f.f.) 369.7 (b.f.)	0.1 (f.f.) 0.5 (b.f.)	0.1 (f.f.) 6.5 (b.f.)	0.7 (f.f.) 24.9 (b.f.)	n.r.	0.1 (f.f.) 1.5 (b.f.)	n.r.	n.r.	n.r.	n.r.	811.4	[68]
		6.7 (f.f.) 1093.6 (b.f.)	0.3 (f.f.) 1.5 (b.f.)	0.2 (f.f.) 13.5 (b.f.)	7.1 (f.f.) 75.9 (b.f.)	n.r.	0.6 (f.f.) 5.8 (b.f.)	n.r.	n.r.	n.r.	n.r.	n.r.	[68]
		533.5	n.r.	20.4	89.2	7.0	1.3	n.r.	2.8	n.r.	n.r.	654.2	[76]
	bran	1.0 (f.f.) 208.3 (b.f.)	0.1 (f.f.) 0.5 (b.f.)	0.1 (f.f.) 4.4 (b.f.)	3.7 (f.f.) 13.4 (b.f.)	n.r.	0.1 (f.f.) 1.5 (b.f.)	n.r.	n.r.	n.r.	n.r.	608.1	[68]
*T. turgidum* subsp. *turanicum* (Jakubz.)	grain	6.8 (f.f.) 1571.1 (b.f.)	0.1 (f.f.) 3.2 (b.f.)	0.2 (f.f.) 20.8 (b.f.)	6.1 (f.f.) 59.7 (b.f.)	n.r.	0.6 (f.f.) 10.9 (b.f.)	n.r.	n.r.	n.r.	n.r.	n.r.	[68]
		551.5	n.r.	27.0	124.4	6.2	1.4	n.r.	3.5	n.r.	n.r.	13.9	[76]
	bran	527.8	n.r.	32.6	120.7	8.4	1.3	n.r.	4.1	n.r.	n.r.	694.9	[76]
*T. turgidum* subsp. *polonicum* (L.) Thell.	grain	608.1	n.r.	22.4	69.2	6.9	1.2	n.r.	5.1	n.r.	n.r.	712.8	[76]
*T. turgidum* subsp. *turgidum*	grain	491.0	n.r.	27.5	104.7	7.2	2.6	n.r.	3.1	n.r.	n.r.	635.2	[76]
*T. turgidum* subsp. *carthlicum*	grain	1.2 (f.f.) 310.7 (b.f.)	0.4 (f.f.) 1.1 (b.f.)	0.1 (f.f.) 11.1 (b.f.)	1.1 (f.f.) 29.5 (b.f.)	n.r.	0.1 (f.f.) 2.3 (b.f.)	n.r.	n.r.	n.r.	n.r.	508.3	[68]
*T. turgidum* subsp. *dicoccum* (Schrank ex Schubler) Thell.	grain	2.9 (f.f.) 1894.7 (b.f.)	0.5 (f.f.) 4.3 (b.f.)	0.1 (f.f.) 43.1 (b.f.)	2.1 (f.f.) 169.3 (b.f.)	n.r.	0.3 (f.f.) 14.8 (b.f.)	n.r.	n.r.	n.r.	n.r.	n.r.	[68]
		503.4	n.r.	26.1	121.9	9.6	3.6	n.r.	5.2	n.r.	n.r.	670.1	[76]
	bran	1.0	≤0.02	0.3	n.r.	n.r.	n.r.	n.r.	n.r.	n.r.	n.r.	1320	[13]
*T. turgidum* subsp. *dicoccoides*	grain	586.2	16.3	37.6	88.1	7.2	2.7	1.3	5.0	n.r.	LOD	649–840	[64]
*T. monococcum* L.	grain	4822–10,170	231–749	407–2861	735–1852	n.r.	532–712	n.r.	72–112	n.r.	n.r.	9030–21,060	[73]
		762.9–764.0	n.r.	47.6–54.1	n.r.	n.r.	n.r.	n.r.	n.r.	n.r.	n.r.	n.r.	[66]
		1.1 (f.f.) 336.4 (b.f.)	1.1 (f.f.) 0.4 (b.f.)	0.1 (f.f.) 4.7 (b.f.)	0.5 (f.f.) 29.0 (b.f.)	n.r.	0.2 (f.f.) 1.5 (b.f.)	n.r.	n.r.	n.r.	n.r.	596.4	[68]
		515.1	n.r.	17.4	32.0	4.9	4.2	n.r.	2.0	n.r.	n.r.	575.6	[69]
		1572.5	55.2	2222.4	36.1	62.4	31.1	5.8	41.8	n.r.	2.4	3836–4221	[64]
		2600	≤0.02	0.50	≤0.005	n.r.	n.r.	n.r.	n.r.	n.r.	n.r.	3100	[13]
	husk	576.5	15.0	70.3	80.4	10.9	3.5	1.6	5.3	n.r.	LOD	743–783	[64]
*T. dicoccum* Schrank (Schuebl)	grain	1407.7	23.3	1640.4	21.1	51.9	21.3	4.5	37.7	n.r.	LOD	3131–3285	[64]
		22.2	0.7	1.2	2.5	n.r.	1.8	n.r.	4.0	n.r.	1.3	395	[65]
	husk	734.0	97.8	9.4	63.2	19.5	65.2	42.7	41.0	12.0	n.r.	n.r.	[25]
*T. dicoccon* Schrank	grain	1800.7	242.3	23.4	155.2	48.4	159.4	104.9	100.8	29.6	n.r.	n.r.	[25]
*T. polonicum*	grain	1455.8	33.1	6.2	71.6	26.1	176.7	38.0	20.6	14.9	n.r.	n.r.	[25]
	bran	3408.0	77.6	14.4	167.6	61.2	413.7	89.0	48.3	34.8	n.r.	n.r.	[25]
*T. turanicum*	grain	471.1–724.9	n.r.	102.1	n.r.	n.r.	n.r.	n.r.	n.r.	n.r.	n.r.	n.r.	[77]
	bran	1007.6	n.r.	9.2	20.6	7.2	3.5	n.r.	12.3	n.r.	n.r.	1060.5	[69]
*T. urartu* Tum.	grain	779.0	n.r.	10.1	30.3	7.5	3.2	n.r.	8.2	n.r.	n.r.	838.3	[69]
*T. sphaerococcum* *Percival*	grain												
*T. carthlicum Nevski*	grain												

LOD—below the limit of detection; f.f.—free form; b.f.—bound form; n.r.—not researched; n.d.—not detected.

**Table 2:** Flavonoids in some wheat species.

Wheat Species/Cultivars	Material	Major Flavonoids	Identification Methods	References
*T. polonicum* (breeding lines) *T. durum* (cv. Duroflavus, Duromax, Floradur, Malvadur) *T. aestivum* (cv. Torka, Zebra, Kontesa, Parabola) *T. turanicum* (Kamut^®^ wheat)	Grain	quercetin, naringenin, vitexin, rutin, apigenin, catechin, kaempferol, luteolin	UPLC	[25]
*T. aestivum*	Germ	72 flavone C-glycosyl derivatives (2 mono-C-glycosides, 34 di-C-glycosides, 15 tri-glycosides, 14 acyl di-C-glycosides, 7 acyl tri-glycosides, 7 acylated mono-O-glycosyl-di-C-glycosyl flavones, acylated di-C-glycosyl flavones): apigenin and luteolin derivatives	UPLC-PDA-ESI/HRMS^n^ + MDF	[115]
Eight wheat cultivars (Fu xilv, Zhongmai 175, Jimai 23, Jimai 22, Taikeheimai 1, Hei jingang, Bainong 307, and Jimai 44) in China	Sprouts	dihydroquercetin, quercetin, vitexin, dihydrokaempferol, rutin, quercitrin, naringenin, kaempferol, apigenin	HPLC-DAD	[116]
Oriental wheat (Khorasan wheat; *T. turgidum* ssp. *turanicum*)	Sprouts	11 flavonoid *C*-glycosides (apigenin and luteolin derivatives), chrysoeriol-8-*C*-glucoside-2″-*O*-arabinoside, chrysoeriol-8-*C*-glucoside-2″-*O*-rhamnoside	UHPLC-QTOF MS	[117]
*T. aestivum*	Sprouts	isoschaftoside, isoorientin, isoscoparin, other C-/O-glycosylated flavones (14 compounds)	HPLC-DAD-ESI-MS	[118]
Winter wheat (*T. aestivum*) (cv. HPm512, ZM22)	Grain	apigenin glycosides (apigenin-6-*C*-(2″-glucosyl)-arabinoside and apigenin-8-*C*-(2″-glucosyl)-arabinoside), luteolin glycosides (luteolin-6-*C*-glucoside), tricin and seven tricin glycosides, kaempferol glycosides, quercetin glycosides	UPLC-MS/MS	[107]
Durum wheat (*T.* *durum* Desf.) landraces (Biancuccia, Margherito, Manto di Maria, Ruscia, Russello SG8, Scavuzza, Tumminia SG3, Trentino, Tripolino, Urria), Durum wheat (cv Core, Mimmo, Simeto)	Grain	flavone-C-glycoside (apigenin and luteolin derivatives)	HPLC-ESI-MS	[108]
Durum wheat (*T. turgidum* *L.* var. *durum*)	Grain	derivatives of apigenin (apigenin- 6-*C*-arabinoside-8-*C*-hexoside and apigenin-6-*C-β*-galactosyl-8-*C*-β-glucosyl-*O* glucuronopyranoside)	LC-ESI-QTOF-MS	[110]
Durum wheat (*T. turgidum* ssp. *Durum*) (cv. Chili, Jneh Khotifa, Karim, Maali, Maghrebi, Om Rabiaa, Razzek)	Grain	apigenin derivatives (apigenin-6- *C-β*-galactosyl-8-*C-β*-glucosyl-*O*-glucopyranoside=schaftoside and apigenin-6-*C*-arabinoside-8-*C*-hexoside= isoschaftoside), quercetin-3,7-di-*O*-glucoside	UPLC-ESI-QTOF-MS/MS	[109]
Bread wheat (*T. aestivum*) *T. monococcum, T. uratu,* *T. durum, T. spelta,* *T. dicoccum, T. polonicum,* *T. turgidum,* *T.diccoides, T. carthlicum*	Grain	apigenin di-C-glycosides (ACGs)	HPLC	[111]
Common wheat (*T. aestivum*)/(cv. JM20) Black wheat *(T. aestivum*)/(cv. HJ1) Green wheat (*T. aestivum*)/(cv. HZ148)	Grain	peonidin-3-(6-*O-p*-coumaroyl)-glucoside cyanidin-3-*O*-(6-*O*-malonyl-*β-D*-glucoside), cyanidin-*3-O*-glucoside, peonidin-*3-O*-glucoside, naringin, procyanidin B3 delphinidin-*3-O-*glucoside, delphinidin-*3-O*-sambubioside, cyanidin-*3-O*-glucoside, procyanidin B3	UPLC-MS/MS	[114]

**Table 3:** Coumarin content of some wheat species.

Wheat Species/Cultivars	Coumarin Type	Content (mg/kg)	References
*T. aestivum*/Ebi (CHR—site, 1999—year, control)	4-hydroxycoumarin	0.1	[123]
*T. aestivum*/Ebi (CHR—site, 1998/1999—year, control)	7-hydroxycoumarin (umbelliferone) Coumarin	133/32.6 0.7/0.5	[123]
*T. aestivum*/Ebi (JAR—site, 1998/1999—year, control)	7-hydroxycoumarin Coumarin	88.6/29.7 0.5/0.8	
*T. aestivum*/Ebi (STA—site, 1998/1999—year, control)	7-hydroxycoumarin Coumarin	15.2/24.2 0.6/1.1	
*T. aestivum*/Estica (CHR—site, 1998/1999—year, control)	7-hydroxycoumarin Coumarin	92.7/28.2 0.8/1.1	[123]
*T. aestivum*/Estica (JAR—site, 1998/1999—year, control)	7-hydroxycoumarin Coumarin	109/126 1.2/1.6	
*T. aestivum*/Estica (STA—site, 1999—year, control)	7-hydroxycoumarin Coumarin	104 1.4	
*T. aestivum*/Nela (CHR—site, 1999—year, control)	7-hydroxycoumarin Coumarin	17.6 0.7	[123]
*T. aestivum*/Nela (JAR—site, 1998/1999—year, control) *T. aestivum*/Nela (JAR—site, 1999—year, control)	7-hydroxycoumarin Coumarin	71.7/51.5 1.7	
*T. aestivum*/Samanta (CHR—site, 1998/1999—year, control) *T. aestivum*/Samanta (CHR—site, 1999—year, control)	7-hydroxycoumarin Coumarin	42.1/11.2 0.6	[123]
*T. aestivum*/Samanta (JAR—site, 1998/1999—year, control) *T. aestivum*/Samanta (JAR—site, 1999—year, control)	7-hydroxycoumarin Coumarin	40.2/28.6 1.9	
*T. aestivum*/Samanta (STA—site, 1998/1999—year, control) *T. aestivum*/Samanta (STA—site, 1999—year, control)	7-hydroxycoumarin Coumarin	114/21.3 2.2	
*T. aestivum*/Šárka (CHR—site, 1999—year, control)	7-hydroxycoumarin Coumarin	19.6 0.8	[123]
*T. aestivum*/Šárka (JAR—site, 1998/1999—year, control) *T. aestivum*/Šárka (JAR—site, 1999—year, control)	7-hydroxycoumarin Coumarin	73.1/33 0.8	
*T. aestivum*/Šárka (STA—site, 1998/1999—year, control)	7-hydroxycoumarin Coumarin	15.3/20.5 1.5/0.8	
Durum wheat (*T. turgidum* L. var. *durum*)/Bousselam, Vitron, Gaviota durum	Coumarins (class)	present (screening test)	[124]
Durum wheat *(T. durum* Desf.*)* Soft wheat (*T. aestivum* L.)	Coumarins (class)	present (screening test)	[125]
Common wheat *(T. aestivum* L.*)*/Gentil Rosso Mutico, Marzuolo d’Aqui	Coumarin (bound extract)	Present (HPLC-ESI-TOF-MS)	[5]

**Table 4:** AR content (μg/g) in different *Triticum* species.

*Triticum* Species	Material	Total ARs	References
*T. timophevi*	grain	1480	[29]
*T. dicoccum*	grain	1148	[140]
		843	[142]
		422–737	[64]
		444–581	[134]
	husk	73–226	[64]
*T. aestivum*	grain	680–1138	[62]
		591–968	[64]
		957	[142]
		909	[29]
		494–655	[138]
		340–416	[134]
		255	[140]
*T. compactum*	grain	1090	[29]
*T. ispahanicum*	grain	982	
*T. polonicum*	grain	951	
*T. monococcum*	grain	925	[140]
		770	[142]
		399–595	[134]
		400–456	[64]
		391	[141]
	husk	51–217	[64]
*T. paleocolchicum*	grain	916	[29]
*T. spelta*	grain	712–844	[62]
		818	[142]
		470–782	[64]
		427–605	[134]
		455	[139]
	husk	267–515	[64]
*T. durum*	grain	687	[29]
		370–452	[138]
		444	[141]
		327–399	[134]
		191	[140]
*T. araraticum*	grain	599	[29]
*T. turanicum*	grain	200	
		256	[141]

**Table 5:** Comparative carotenoid composition of some wheat species.

Wheat Species/Type	Lutein (µg/g)	*β*-Carotene (µg/g)	Zeaxanthin (µg/g)	Total Carotenoids (µg/g)	References
Durum (*T. turgidum* ssp. *durum*)	5.40	0.21	0.56	6.17	[165]
Bread wheat (*T. aestivum*)	3.79	0.19	0.67	4.65	
Emmer (*T. dicoccum*)	3.76	0.23	0.46	4.46	
Spelt (*T. spelta*)	2.84	0.25	0.41	3.46	
*T. polonicum*	1.18	1.36	0.63	3.17	[25]
*T. durum*	1.92	2.04	0.89	4.84	
*T. aestivum*	1.56	1.59	0.79	3.94	
*T. turanicum* (Kamut)	1.35	1.45	0.81	3.61	
Indian dwarf (*T. sphaerococcum Percival*)	1.16	0.05	0.18	1.45	[69]
Persian wheat (*T. carthlicum Nevski*)	1.35	0.06	0.14	1.64	
Bread wheat (cv. Zyta)	1.95	0.04	0.17	2.32	
Durum wheat (cv. Komnata),	3.04	0.05	0.2	3.58	
Spelt wheat (cv. Schwabenkorn)	3.05	0.05	0.16	3.51	
Einkorn wheat (unknown cv.)	7.03	0.09	0.31	7.92	
184 different durum wheat cultivars (*T. turgidum* ssp. *durum*) in Germany	2.67	0.4	n.r.	3.06	[177]
*T. aestivum* L. (Red standard)	n.r.	n.r.	n.r.	1.61	[176]
*T. aestivum* L. (Blue aleurone)	n.r.	n.r.	n.r.	1.85	
*T. aestivum* L. (Purple pericarp)	n.r.	n.r.	n.r.	1.70	
Yellow endosperm	n.r.	n.r.	n.r.	6.82	
Spanish Durum Wheat Landraces (*T. durum* L.) (*dicoccon* and *turgidum* subspecies)	0.84–4.77 (µg/g FW)	0.01–0.07 (µg/g FW)	0.12–0.48 (µg/g FW)	1.03–5.27 (µg/g FW)	[178]
Commercial durum wheat (*T. durum* L.) cultivars (Amilcar, Kofa, Monastir, and Olivadur)	3.18 (µg/g FW)	0.03 (µg/g FW)	0.23 (µg/g FW)	3.44 (µg/g FW)	
Einkorn (*T. monococcum*)	4.11–12.64	n.d.–0.65	0.29–0.94	5.33–13.64	[87]
Soft winter wheat (*T. aestivum*), modern cultivars)	0.10–0.69	n.d.	0.03–0.18	0.11–0.85	[172]
Einkorn wheat (*T. monococcum* L., subsp. *monococcum*)	5.38	0.53 (α + β)-carotene	0.68	7.29	[43]
Emmer (*T. dicoccum*)	1.38	0.24 (α + β)-carotene	0.43	2.34	
*T. timopheevi*	3.62	0.33 (α + β)-carotene	0,50	4.9	
Georgian emmer (*T. turgidum* L., subsp. *paleocolchicum*)	1.16	0.17 (α + β)-carotene	0.35	2.17	
Durum wheat (*T. turgidum* L., subsp. *durum* (Desf.) Husnot)	3.36	0.44 (α + β)-carotene	0.52	4.75	
Macha wheat (*T. aestivum* L., subsp. *macha*)	2.14	0.19 (α + β)-carotene	0.32742	1.15	
Bread wheat (*T. aestivum*, primitive accessions)	2.29	0.21(α + β)-carotene	0.38	1.19	
Einkorn (*T. monococcum* L., subsp. *monococcum*)	5.246	0.195	0.351	n.r	[170]
Emmer (*T. dicoccum* Schuebl [Schrank], subsp. *dicoccum*)	0.761	n.d.	0.138	n.r.	
Spring bread wheat (*T. aestivum* L.)	1.096	0.116	0.144	n.r	
Spring wheat cultivars (*T. aestivum*) (Alpowa, Blanca Grande, Louise, Macon, and WestBred 936)	1.5–4.0	n.r.	n.r.	n.r.	[171]
Common bread wheat (*T. aestivum*)	2.01–2.11	n.r	n.r	2.89–3.01 (total yellow pigments)	[36]
Durum, Kamut, and Khorasan (*T. turgidum*)	5.41–5.77	n.r	n.r	7.61–8.15 (total yellow pigments)	
Einkorn (*T. monococcum*)	6.37–8.46	n.r	n.r	8.56–13.79 (total yellow pigments)	
Bread wheat, Chinese cultivars (217 total)	0.183–1.00	0.0089–48.7	0.048–0.12	variable	[6]
Bread wheat with 1BL.1RS translocation	0.30	0.23	0.09	0.594	
Einkorn (*T. monococcum*), cv. Monlis—wholemeal	6.75	0.96	0.29	8.09	[87]
Bread wheat (cv. Serio)—wholemeal	0.90	n.d.	0.11	1.01	
Bread wheat (cv. Serio)—white flour	1.03	n.d.	0.07	1.10	
Einkorn—white flour	8.42	0.98	0.27	9.77	
Einkorn (*T. monococcum*)	7.09	0.57	0.27	7.66	[167]
Maryland Soft Wheat (range)	0.82–1.14	0.10–0.21	0.20–0.39	1.12–1.74	[46]

n.r.—not researched; n.d.—not detected.

## Data Availability

Not applicable.
